# Hypoxic microenvironment in cancer: role in metabolic reprogramming

**DOI:** 10.3389/fonc.2026.1771365

**Published:** 2026-03-31

**Authors:** Niti Sureka, Rashi Maheshwari, Amit Agravat, Shweta Singhal, Shamsuz Zaman, Bhavika Rishi, Fouzia Siraj, Sufian Zaheer, Aroonima Misra

**Affiliations:** 1 Department of Pathology, Vardhman Mahavir Medical College and Safdarjung Hospital, New Delhi, India; 2 Department of Pathology, Pandit Deendayal Upadhyay (PDU) Medical College, Rajkot, India; 3 Department of Anatomy, Vardhman Mahavir Medical College and Safdarjung Hospital, New Delhi, India; 4 Indian Council of Medical Research (ICMR)- Centre for Cancer Pathology, New Delhi, India; 5 ICMR-National Institute of Child Health and Development Research, New Delhi, India

**Keywords:** hypoxia, hypoxia-inducible factors (HIFs), immune evasion, metabolic reprogramming, mitochondrial metabolism, tumor microenvironment (TME), Warburg effect

## Abstract

Hypoxia, a defining hallmark of solid tumors, arises from structurally and functionally abnormal vasculature, rapid cellular proliferation, and impaired perfusion, resulting in chronic and cycling oxygen deprivation within the tumor massThe hypoxic tumor microenvironment orchestrates extensive molecular reprogramming primarily through stabilization and activation of hypoxia-inducible factors (HIF-1α and HIF-2α), which regulate broad transcriptional networks governing metabolism, angiogenesis, stemness, invasion, and immune modulation. Under low oxygen tension, tumor cells shift toward aerobic glycolysis, enhance glutamine utilization, promote lipid synthesis and storage, suppress mitochondrial oxidative phosphorylation, and fine-tune redox balance through coordinated regulation of ROS-generating and antioxidant systems. These adaptations not only sustain proliferation and survival under metabolic stress but also facilitate epithelial–mesenchymal transition, extracellular matrix remodeling, and metastatic dissemination. Beyond malignant cells, hypoxia reprograms stromal compartments—including cancer-associated fibroblasts, endothelial cells, tumor-associated macrophages, and myeloid-derived suppressor cells—thereby establishing a metabolically cooperative, angiogenic, and profoundly immunosuppressive microenvironment. Hypoxia-induced acidosis, lactate accumulation, and HIF-driven cytokine signaling further impair cytotoxic T-cell and NK-cell activity, contributing to immune escape and resistance to radiotherapy, chemotherapy, and immunotherapy. Emerging evidence from single-cell multi-omics, spatial transcriptomics, metabolic imaging, and early-phase clinical trials targeting HIF signaling, angiogenic pathways, and metabolic enzymes has uncovered actionable vulnerabilities in hypoxia-driven malignancies. This review synthesizes the mechanistic foundations of hypoxia-induced metabolic reprogramming, its role in tumor progression and therapeutic resistance, and discusses innovative strategies aimed at exploiting hypoxia-associated metabolic dependencies to advance precision oncology.

## 1 Introduction

The tumor microenvironment (TME) is a complex and dynamic ecosystem composed of cancer cells, stromal elements, immune infiltrates, blood vessels, and extracellular matrix components (1) One of the defining features of the TME is its heterogeneous distribution of oxygen, nutrients, and metabolic byproducts, which creates areas of physiological stress (2). Among these, hypoxia, or a condition of reduced oxygen availability, has emerged as a critical determinant of tumor progression, treatment resistance, and metastatic potential (3, 4). Hypoxia typically arises due to the aberrant and insufficient vascularization within tumors, which is unable to meet the high oxygen demands of rapidly proliferating cancer cells (4).

**Figure 1:**
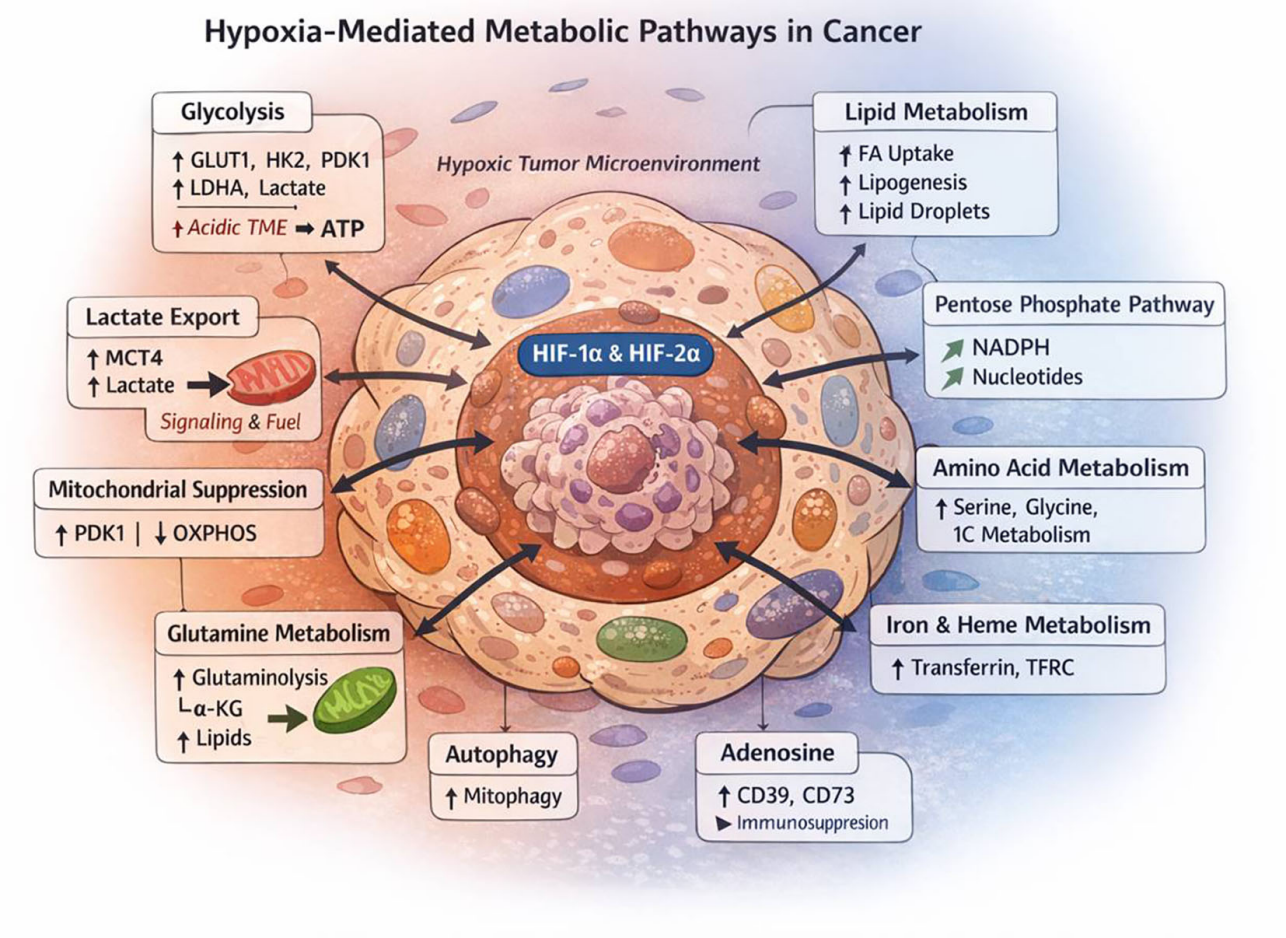
Hypoxia-mediated metabolic reprogramming in cancer cells.This schematic illustrates how hypoxic conditions within the tumor microenvironment stabilize hypoxia-inducible factors (HIF-1α and HIF-2α), which act as central transcriptional regulators orchestrating widespread metabolic adaptations that promote tumor survival, growth, and immune evasion. Under hypoxia, HIF-1α drives a shift toward aerobic glycolysis by upregulating glucose transporters (GLUT1/3) and key glycolytic enzymes (HK2, LDHA), while induction of pyruvate dehydrogenase kinase-1 (PDK1) inhibits pyruvate entry into the tricarboxylic acid (TCA) cycle, thereby suppressing mitochondrial oxidative phosphorylation (OXPHOS). Excess pyruvate is converted to lactate, which is exported via monocarboxylate transporter-4 (MCT4), contributing to extracellular acidification, metabolic symbiosis, and immunosuppression. In parallel, hypoxia enhances glutamine metabolism, particularly through HIF-2α-dependent pathways, supporting anaplerosis, reductive carboxylation, and lipid biosynthesis. Lipid metabolic reprogramming is characterized by increased fatty acid uptake, de novo lipogenesis, and lipid droplet accumulation, which provide energy reserves and protect against oxidative stress. Increased flux through the pentose phosphate pathway (PPP) generates NADPH and nucleotides, maintaining redox homeostasis and supporting biosynthetic demands. Hypoxia also modulates amino acid metabolism, notably serine–glycine–one-carbon pathways, to sustain nucleotide synthesis and redox balance. Additionally, HIF-regulated iron and heme metabolism ensures iron availability for essential cellular processes.

**Figure 2:**
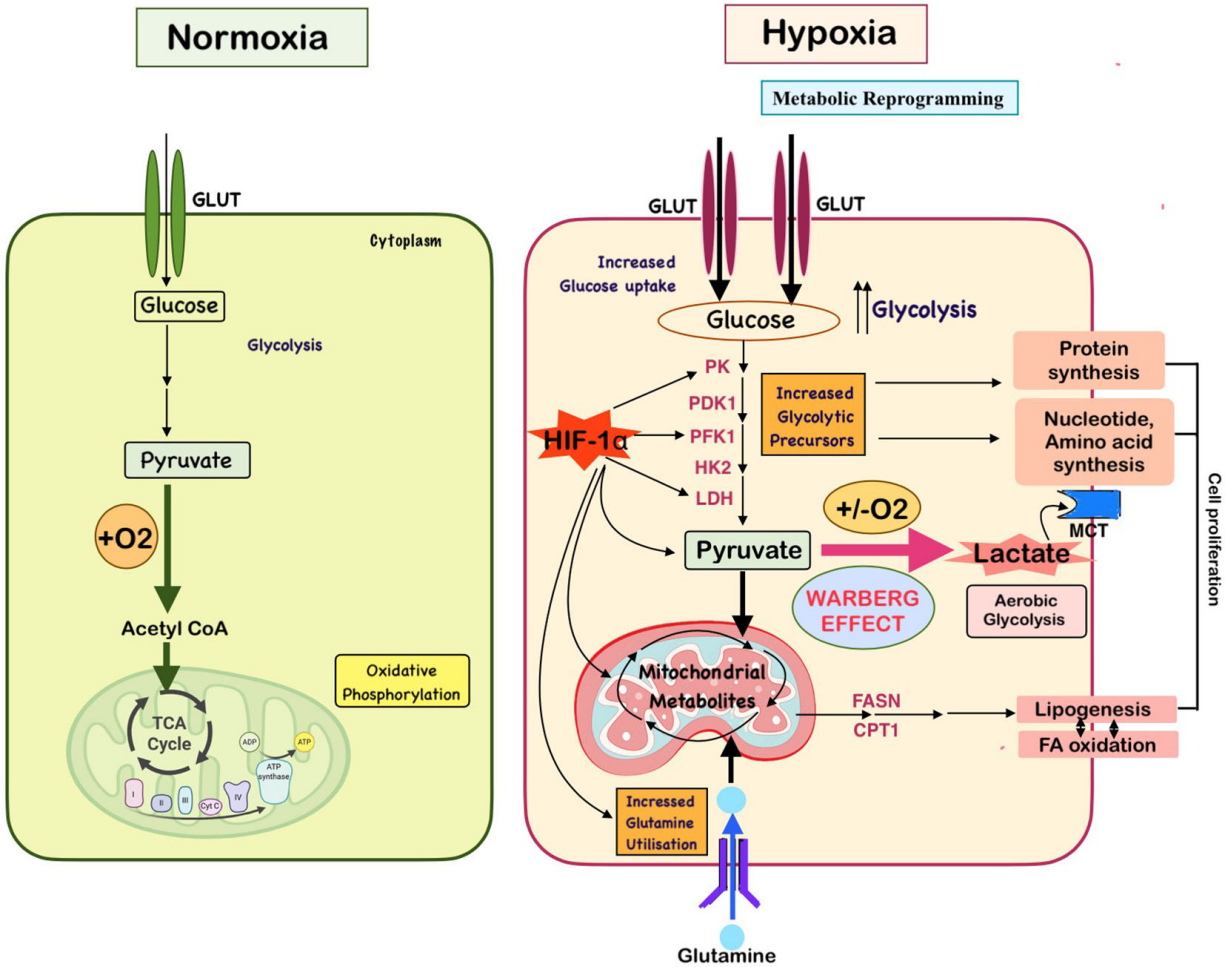
Metabolic reprogramming in the hypoxic tumor microenvironment drives cancer aggressiveness. Under normoxic conditions, tumor cells primarily depend on mitochondrial oxidative phosphorylation for ATP generation. In contrast, under low oxygen conditions, HIFs initiate a cascade of transcriptional changes that promote metabolic adaptation. Key features include a glycolytic shift (Warburg effect), characterized by increased glucose uptake, enhanced glycolysis, elevated lactate export and glutamine metabolism to support biosynthesis and reprogramming of lipid metabolism, to support membrane synthesis and energy needs. Additionally, suppression of mitochondrial oxidative metabolism contributes to redox homeostasis. [FASN: fatty acid synthase; CPT1A: carnitine palmitoyltransferase; PDK1: pyruvate dehydrogenase kinase 1; PK – Pyruvate Kinase; PFK – Phosphofructokinase; LDH – Lactate Dehydrogenase; LDH2 – Lactate Dehydrogenase Isoenzyme 2; MCT – Monocarboxylate Transporter.]

This oxygen deficiency activates a range of cellular adaptive mechanisms, orchestrated primarily through hypoxia-inducible factors (HIFs), particularly HIF-1α and HIF-2α (**Figure 1**) (5–7). These transcription factors regulate the expression of genes involved in angiogenesis, glycolysis, pH regulation, erythropoiesis, and cell survival (8, 9). Through these HIF-mediated pathways, hypoxic tumors undergo metabolic reprogramming, a hallmark of cancer, enabling cells to maintain ATP production, reduce oxidative stress, and thrive under nutrient-limiting conditions (10, 11). Notably, this shift includes increased reliance on anaerobic glycolysis (the Warburg effect), enhanced glutamine metabolism, alterations in fatty acid oxidation, and remodeling of mitochondrial function (**Figure 2**) (11).

Importantly, hypoxia-induced metabolic reprogramming not only promotes cancer cell survival but also actively contributes to immune evasion, therapeutic resistance, and a more aggressive phenotype (10, 12). Hypoxic regions within tumors are often associated with necrosis, acidosis, and recruitment of immunosuppressive cells such as tumor-associated macrophages (TAMs) and myeloid-derived suppressor cells (MDSCs) (10, 13). This adaptation also influences the behavior of non-malignant components of the TME, reinforcing a supportive niche for tumor progression.

Recent advances in multi-omics, single-cell analysis, and imaging technologies have deepened our understanding of the hypoxic tumor landscape and revealed novel metabolic vulnerabilities (14, 15). Consequently, targeting the hypoxic response and the associated metabolic pathways has gained considerable interest as a therapeutic strategy (16).

This review provides a comprehensive overview of the hypoxic microenvironment in cancer, with a particular focus on its role in metabolic reprogramming. We discuss the molecular mechanisms underpinning hypoxia-driven metabolic adaptations, highlight their implications for tumor progression and therapy resistance, and explore emerging strategies aimed at disrupting these processes to improve cancer treatment outcomes.

## 2 Mechanisms of hypoxia in the tumor microenvironment

### 2.1 Development of hypoxia in tumors

**Figure 3:**
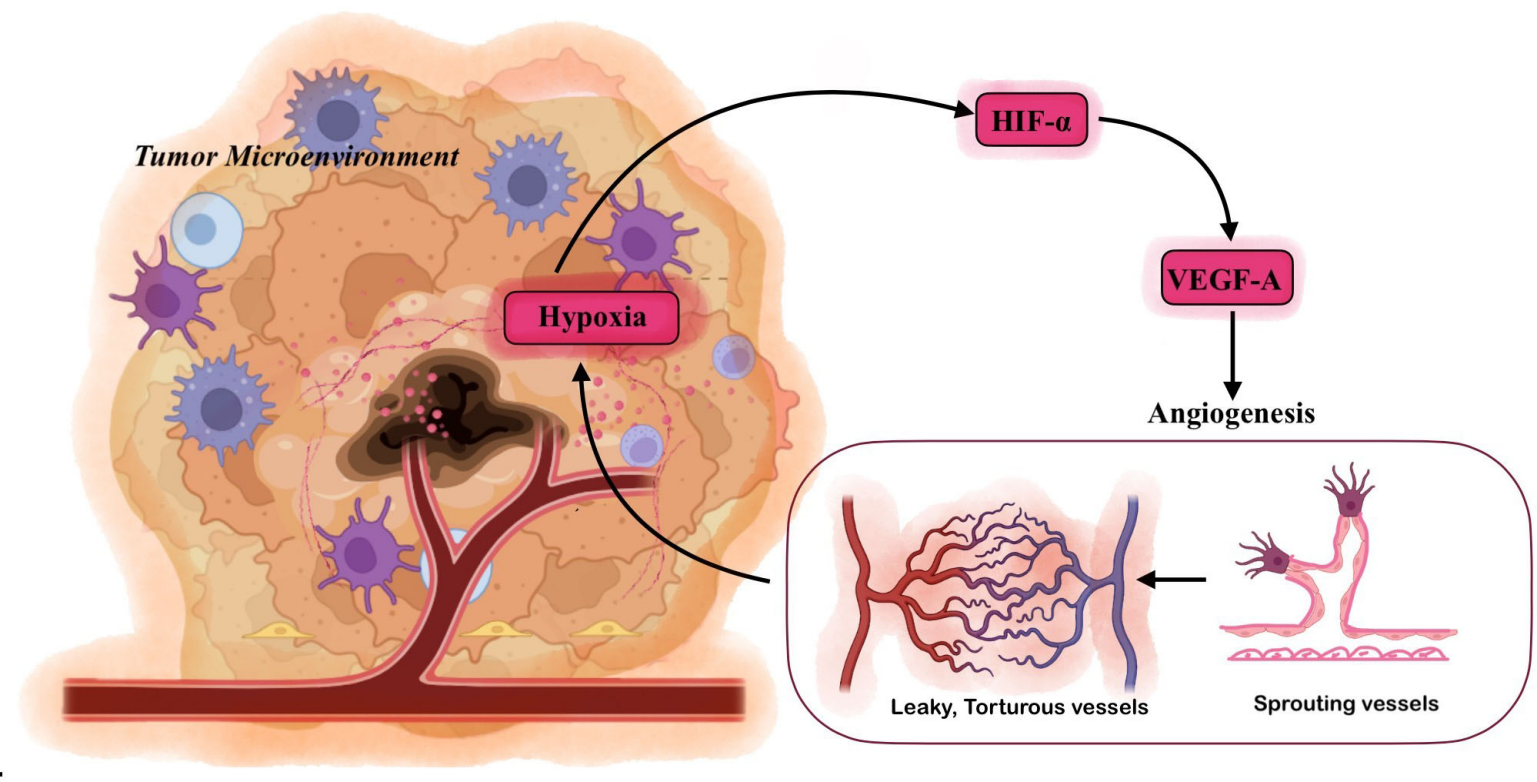
Hypoxia-Induced Angiogenesis in the Tumor Microenvironment: Within the hypoxic tumor microenvironment, reduced oxygen availability stabilizes HIF-1α, which transcriptionally upregulates pro-angiogenic factors such as vascular endothelial growth factor (VEGF). VEGF promotes the angiogenic cascade, which involves endothelial cell activation, degradation of the basement membrane, and the sprouting of new capillary vessels toward hypoxic regions. However, due to the uncoordinated and excessive nature of tumor angiogenesis, the newly formed vessels are tortuous, dilated, leaky, and poorly organized, resulting in inefficient perfusion and perpetuation of hypoxia.

Hypoxia is a common feature observed in approximately 90% of solid tumors and is widely recognized as a hallmark of cancer (17). The emergence of hypoxia within the tumor microenvironment is primarily a consequence of the rapid and disorganized proliferation of malignant cells, which often surpasses the capacity of the existing vascular network to supply adequate oxygen (10, 17). The concept of tumor hypoxia was first introduced by Thomlinson and Gray in 1955, when they described a declining oxygen gradient extending from the outer regions toward the center of the tumor mass (18). Tumors typically induce angiogenesis through the secretion of pro-angiogenic factors such as vascular endothelial growth factor (VEGF), aiming to meet their increasing metabolic demands (19, 20). However, the newly formed blood vessels are frequently aberrant in structure and function—they are irregularly shaped, dilated, leaky, and poorly perfused (**Figure 3**) (21). These abnormalities contribute to heterogeneous oxygen distribution, creating regions of low oxygen tension within the tumor mass (21).

Hypoxia in tumors can be broadly categorized into two distinct forms: chronic hypoxia and acute (or cycling) hypoxia (22). Chronic hypoxia develops as a result of the limited diffusion capacity of oxygen from functional blood vessels into the inner regions of expanding tumor masses for prolonged period (>24 h) (4, 22). Oxygen can typically diffuse only 100–200 micrometers from a capillary; beyond this threshold, cells experience sustained oxygen deprivation (10). This form of hypoxia is relatively stable and persists over time, leading to long-term cellular adaptation via transcriptional reprogramming (3, 10).

In contrast, acute hypoxia, also referred to as cycling or intermittent hypoxia, arises due to transient fluctuations in tumor blood flow ((no longer than 24 h))., leading to to oxygen levels below 1% that can be reversed with returned blood flow (22). These fluctuations are caused by the chaotic nature of tumor vasculature and the dynamic changes in vessel patency, including intermittent vessel collapse, thrombosis, or vasoconstriction (22). As a result, regions within the tumor may alternate between normoxic and hypoxic states over short periods. This cycling hypoxia imposes repeated stress on cancer cells and selects for phenotypes that are highly adaptable and resistant to apoptosis, promoting tumor aggressiveness and therapeutic resistance (13, 23). Brown JM provided a critical experimental evidence supporting the existence of acutely hypoxic cells in solid tumors of mice. Unlike chronically hypoxic cells, which experience long-term oxygen deprivation due to limitations in diffusion, acutely hypoxic cells were shown to arise from transient fluctuations in blood flow within the tumor microenvironment. Brown proposed that this temporary hypoxia results from the intermittent perfusion of tumor blood vessels, a consequence of the abnormal and disorganized tumor vasculature (24).

Both chronic and acute hypoxia coexist within tumors and play critical, yet distinct, roles in shaping the tumor biology. While chronic hypoxia drives long-term metabolic reprogramming and stable epigenetic alterations (10, 25, 26), acute hypoxia promotes oxidative stress, genomic instability, and selective pressures that favor more malignant phenotypes (10, 27).

### 2.2 Hypoxia-inducible factors

**Figure 4:**
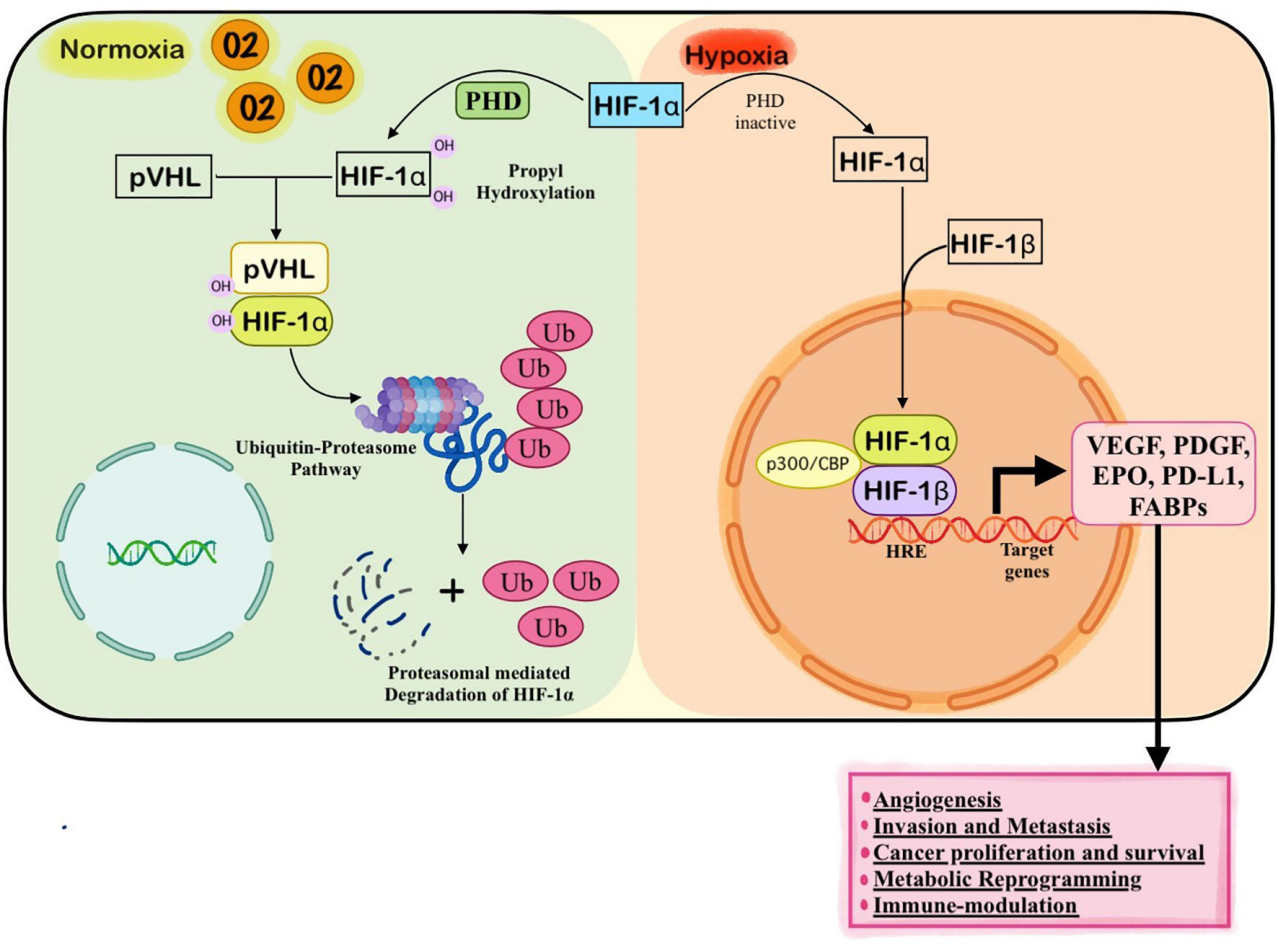
Regulation of HIF-1α by Oxygen Levels under Normoxic and Hypoxic Conditions. Under normoxic conditions (left panel), HIF-1α is hydroxylated by prolyl hydroxylase domain (PHD) enzymes in an oxygen-dependent manner. This hydroxylation targets HIF-1α for recognition by the von Hippel-Lindau (VHL) E3 ubiquitin ligase complex, leading to its ubiquitination and subsequent degradation by the proteasome, thereby maintaining low intracellular HIF-1α levels and preventing transcriptional activation. In contrast, under hypoxic conditions (right panel), PHD enzymes are inactivated due to the lack of oxygen, allowing HIF-1α to accumulate and translocate into the nucleus. There, it dimerizes with HIF-1β and binds to hypoxia-response elements (HREs) on DNA, recruiting co-activators such as CBP/p300 to initiate the transcription of hypoxia-responsive genes like VEGF and EPO, facilitating cellular adaptation to low oxygen.

**Figure 5:**
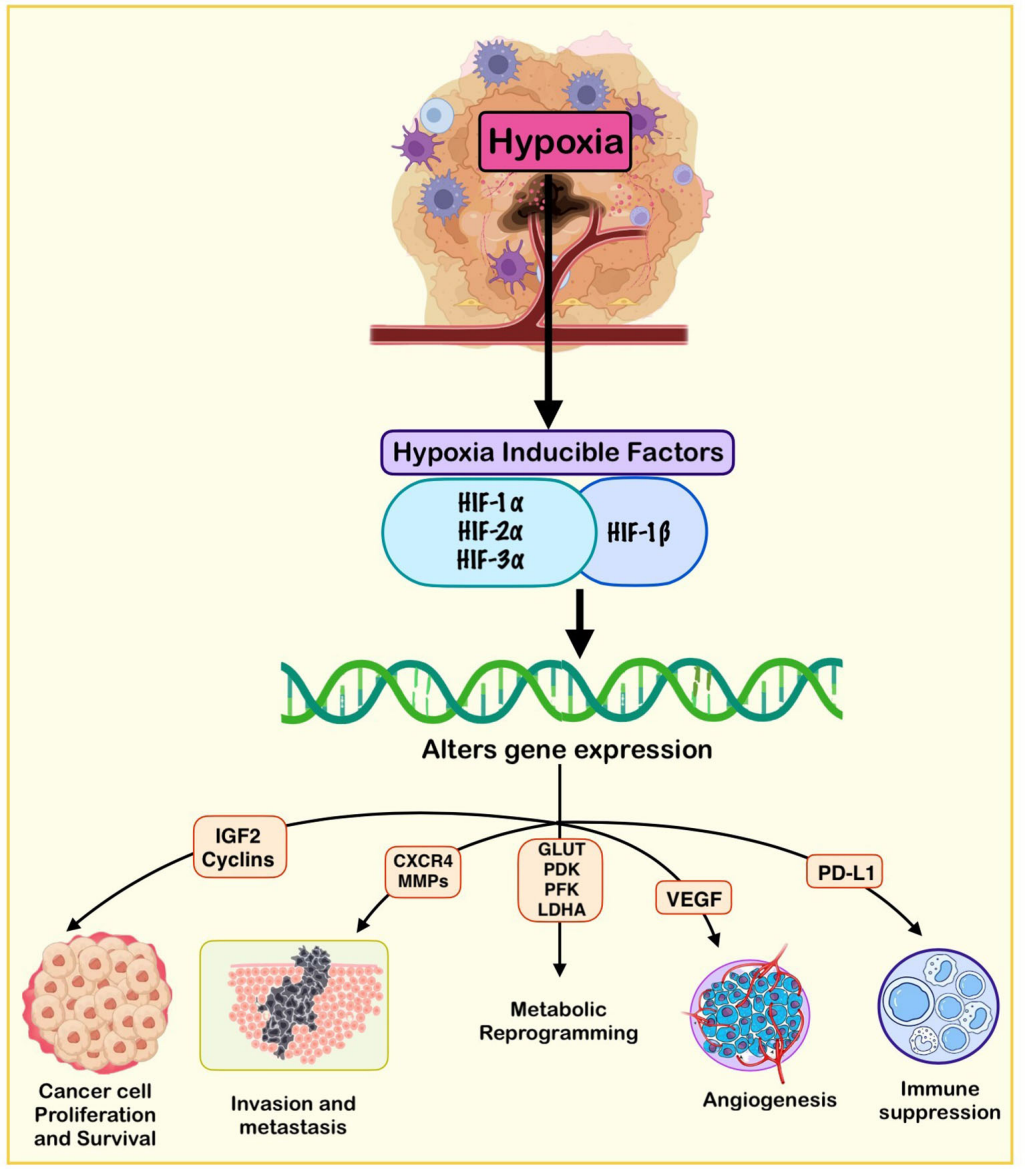
Mechanism of HIF-1 in Hypoxic Tumors. In the hypoxic tumor microenvironment, oxygen deprivation stabilizes hypoxia-inducible factors (HIF-1α, HIF-2α, and HIF-3α) and their constitutively expressed partner HIF-1β. The active HIF complexes translocate to the nucleus and induce the transcription of a wide array of target genes that promote tumor progression. These include genes regulating cancer cell proliferation and survival (e.g., IGF2, Cyclin D1), epithelial–mesenchymal transition and metastasis (e.g., CXCR4, MMPs), metabolic reprogramming (e.g., GLUT1, LDHA, HK2), angiogenesis (e.g., VEGF, PDGF), and immunosuppression (e.g., PD-L1).

Hypoxia-inducible factors (HIFs) are central regulators of the cellular response to low oxygen tension and play a pivotal role in mediating the adaptive changes observed in hypoxic tumors (6). These transcription factors enable cancer cells to survive and proliferate under oxygen-deprived conditions by altering gene expression profiles that influence metabolism, angiogenesis, erythropoiesis, cell survival, and immune modulation (7, 28). HIFs are heterodimeric proteins composed of an oxygen-regulated α-subunit (HIF-1α, HIF-2α, or HIF-3α) and a constitutively expressed β-subunit (HIF-1β, also known as ARNT) (**Figure 4**) (8). Among these, HIF-1α is the most extensively studied and is considered the master regulator of the hypoxic response in solid tumors (8). Under normoxic conditions (**Figure 5**), HIF-1α is continuously synthesized but rapidly degraded through the oxygen-dependent activity of prolyl hydroxylase domain (PHD) enzymes, which hydroxylate specific proline residues (Pro-564 and Pro-402) within the oxygen-dependent degradation domain (ODD) of the HIF-1α subunit. This hydroxylation serves as a recognition motif for the von Hippel-Lindau (VHL) E3 ubiquitin ligase complex, leading to ubiquitination and subsequent proteasomal degradation of HIF-1α (29, 30). Thus, in the presence of sufficient oxygen, the intracellular levels of HIF-1α remain low and transcriptional activation of hypoxia-responsive genes is suppressed. However, under hypoxic conditions (**Figure 4**), the activity of PHD enzymes is inhibited due to the lack of oxygen, an essential co-substrate for their enzymatic function (31). As a result, HIF-1α escapes hydroxylation and degradation, accumulates in the cytoplasm, and translocates to the nucleus, where it dimerizes with HIF-1β. The HIF-1α/β heterodimer binds to hypoxia-response elements (HREs) within the promoter regions of target genes and recruits co-activators such as CBP/p300 to initiate transcription (32). This activation triggers a broad transcriptional program that facilitates cellular adaptation to hypoxia (29).

**Figure 6:**
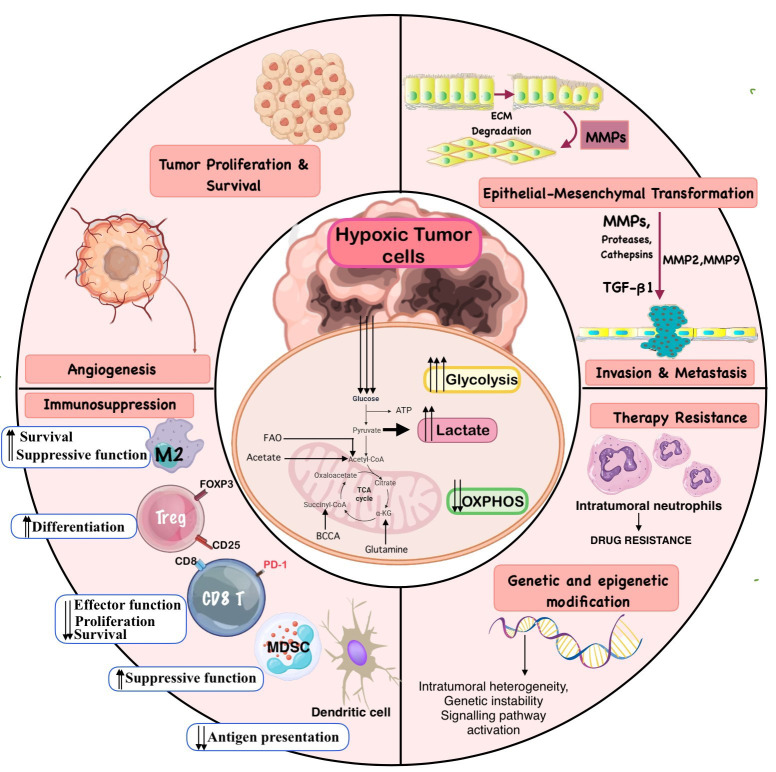
Implications of Metabolic Reprogramming in the Tumor Microenvironment. The central hypoxic tumor cell undergoes metabolic reprogramming characterised by increased glycolysis, elevated lactate production, and reduced oxidative phosphorylation (OXPHOS). These metabolic alterations lead to the accumulation of acidic metabolites and promote survival under oxygen-deprived conditions. Increased glycolysis supports cancer cell survival and proliferation in hypoxic niches by generating ATP anaerobically and producing lactate. The extracellular accumulation of lactate and resulting acidification promote extracellular matrix degradation and epithelial-to-mesenchymal transition (EMT), aiding invasion and metastasis. The acidic and lactate-rich microenvironment also suppresses cytotoxic T cells and NK cells, impairs dendritic cell function, and favors the recruitment of immunosuppressive regulatory T cells (Tregs) and tumor-associated macrophages (TAMs), promoting immune evasion. Persistent hypoxia and metabolic stress drive therapy resistance through adaptive genetic and epigenetic modifications, enhancing tumor cell plasticity and promoting survival against anticancer treatments.

The downstream effects of HIF activation are extensive and include upregulation of various genes and pathways (**Figure 6**), involved in glycolysis, lactate shuttling & acid-base balance, Angiogenesis & Vascular Remodeling, erythropoiesis & iron metabolism, cell survival & stress response, lipid & amino acid metabolism and Immune Modulation (33–35). Through these targets, HIFs promote a metabolic shift from oxidative phosphorylation to glycolysis, enhance glucose uptake, stimulate new blood vessel formation, and modulate the pH of TME—all of which are critical for sustaining tumor cell viability under hypoxic stress (33). Additionally, HIFs influence the tumor immune landscape by modulating cytokine production, upregulating immune checkpoint molecules, and recruiting immunosuppressive cells, thereby contributing to immune evasion.

Although HIF-2α shares structural similarity with HIF-1α, it governs a distinct yet partially overlapping repertoire of target genes (8). Both isoforms possess comparable domain architecture—including the basic helix-loop-helix (bHLH), PAS, oxygen-dependent degradation (ODD), and transactivation domains (TAD) —and exhibit approximately 48% amino acid sequence identity. They are similarly regulated by proline and asparagine hydroxylation. Despite these similarities, their genomic binding patterns differ: HIF-1α preferentially associates with promoter-proximal regions, whereas HIF-2α predominantly binds to distal enhancer elements (8). While HIF-1α is primarily involved in the acute response to hypoxia, HIF-2α contributes more prominently to chronic adaptations, such as erythropoiesis, angiogenesis, and lipid metabolism (36, 37). Although once believed to have similar functions, growing evidence reveals that HIF-1α and HIF-2α can drive distinct, and sometimes even divergent, biological responses within the same cell type (38). The relative roles and dominance of HIF-1α versus HIF-2α may vary depending on tumor type, oxygen levels, and microenvironmental cues (8). HIF-1α drives the classic metabolic shift to glycolysis—upregulating GLUTs, hexokinases, LDH, etc.—suitable for immediate adaptation. HIF-2α steers long-term adaptations such as lipid metabolism, storage, and oxidative homeostasis (8, 39). During angiogenesis, HIF-1α dominates early (EC proliferation, matrix degradation), whereas HIF-2α supports maturation (pericyte recruitment, vessel stabilization, extracellular matrix reinforcement). The “HIF switch” from acute to chronic hypoxia is mediated by isoform-specific regulators (e.g., HAF/SART1), differential mRNA stability, and ubiquitin–proteasome dynamics, all of which contribute to the preferential stabilization and activity of HIF-2α during prolonged hypoxic conditions (8).

In contrast to HIF-1α and HIF-2α, the biological functions of hypoxia-inducible factor-3α (HIF-3α) are less well characterized and appear to be more context-dependent (40, 41). HIF-3α is encoded by the *HIF3A* gene and undergoes extensive alternative splicing, generating multiple isoforms with divergent transcriptional activities (42). Many HIF-3α variants lack a full transactivation domain, enabling them to function as dominant-negative regulators of HIF-1α and HIF-2α by competing for HIF-1β (ARNT) binding or hypoxia-response element (HRE) occupancy (32, 43). Through this mechanism, HIF-3α can fine-tune hypoxia signaling and prevent excessive or prolonged activation of canonical HIF pathways.

Among the best-studied HIF-3α isoforms is inhibitory PAS domain protein (IPAS), which suppresses hypoxia-induced transcription by sequestering HIF-1α and blocking HIF-1α/β heterodimer formation (44–46). In several cancer models, IPAS expression has been associated with reduced angiogenesis and attenuated tumor growth, highlighting a potential tumor-suppressive role for specific HIF-3α variants (44). However, emerging evidence suggests that not all HIF-3α isoforms are inhibitory. Full-length HIF-3α can activate a distinct subset of hypoxia-responsive genes involved in metabolism, epithelial–mesenchymal transition (EMT), and cellular survival, suggesting isoform-specific pro-tumorigenic functions under certain microenvironmental conditions. Villareal et al. demonstrated that HIF-3α1 plays a pro-metastatic role in colorectal cancer (CRC) by promoting epithelial-to-mesenchymal transition (EMT) and enhancing iron uptake. The study shows that HIF-3α1 is upregulated in hypoxic CRC cells and liver metastases, where it transcriptionally regulates EMT-associated genes, leading to increased cellular plasticity, migration, and invasiveness. Concurrently, HIF-3α1 augments iron metabolism by upregulating key iron transport and storage pathways, thereby supporting metabolic adaptation and survival of metastatic cells within the liver microenvironment. Functional *in vitro* and *in vivo* models confirm that HIF-3α1 drives liver metastatic colonization, while its suppression reduces metastatic burden (47). Zhou et al. reported that HIF-3α functions as a key driver of metastatic behavior in pancreatic cancer by transcriptionally activating the RhoC–ROCK1 signaling pathway. Under hypoxic conditions, HIF-3α expression is markedly increased and directly upregulates RhoC, leading to activation of downstream ROCK1 signaling. This axis promotes cytoskeletal remodeling, enhanced cell motility, invasion, and metastatic potential of pancreatic cancer cells. Functional assays demonstrate that HIF-3α knockdown significantly reduces migration and invasion *in vitro* and suppresses metastasis *in vivo*, while restoration of RhoC signaling rescues these phenotypes. Clinically, elevated HIF-3α correlates with aggressive tumor features and poor prognosis (48).

The role of HIF-3α in cancer is therefore dualistic and highly context-dependent. Heikkilä et al. comprehensively characterized the role of HIF-3α splice variants in cellular hypoxia responses. The study demonstrated that HIF-3α exists as multiple isoforms with distinct structural and functional properties, some of which act as negative regulators of hypoxia signaling by competing with HIF-1α and HIF-2α for DNA binding or dimerization with HIF-β. In contrast, other HIF-3α variants retain transcriptional activity and selectively regulate hypoxia-responsive genes. The authors show that these isoforms are differentially expressed across tissues and hypoxic conditions, indicating context-dependent functions (43). In early or transient hypoxia, HIF-3α may act as a negative feedback regulator, dampening excessive HIF-1α and HIF-2α signaling (49, 50). In contrast, during chronic hypoxia or in specific tumor types—such as pancreatic cancer, lung cancer, and hepatocellular carcinoma—HIF-3α expression has been linked to enhanced tumor aggressiveness, metabolic reprogramming, and resistance to therapy (48, 51, 52). These observations underscore the complexity of HIF-3α biology and suggest that its functional impact is dictated by isoform expression patterns, hypoxic duration, and tumor-specific regulatory networks.

HIF-1β, also known as the aryl hydrocarbon receptor nuclear translocator (ARNT), is the obligate heterodimerization partner for all HIF-α subunits and is indispensable for hypoxia-mediated transcriptional responses (53, 54). Unlike HIF-α subunits, HIF-1β is constitutively expressed and is not regulated by oxygen tension. Its availability and functional integrity are nevertheless critical determinants of HIF transcriptional output (7, 53, 54). Upon stabilization of HIF-α subunits under hypoxia, HIF-1β facilitates nuclear localization, DNA binding, and recruitment of transcriptional co-activators, thereby enabling the activation of hypoxia-responsive gene networks (53–56).

Beyond its canonical role in hypoxia signaling, HIF-1β integrates hypoxic responses with other environmental and metabolic signaling pathways (53, 54). As a shared binding partner of the aryl hydrocarbon receptor (AhR), HIF-1β serves as a molecular node linking hypoxia signaling with xenobiotic metabolism, inflammation, and cellular stress responses. Competition between HIF-α and AhR for HIF-1β can influence tumor behavior, particularly in cancers exposed to environmental toxins or inflammatory cues (57, 58). Dysregulation of HIF-1β expression or function has been associated with altered angiogenesis, metabolic flexibility, and immune modulation within the tumor microenvironment (59).

Although HIF-1β itself is not oxygen-regulated, alterations in its expression levels, post-translational modifications, or subcellular localization can significantly modulate hypoxia-driven tumor biology (60, 61). Reduced HIF-1β availability may limit HIF-α signaling even under hypoxic conditions, whereas increased or stabilized HIF-1β can amplify hypoxia-responsive transcriptional programs, contributing to tumor progression, therapy resistance, and immune evasion (8, 59). Consequently, HIF-1β is increasingly recognized not merely as a passive binding partner but as an active regulator of hypoxic signaling fidelity in cancer.

## 3 Hypoxia-driven metabolic reprogramming

### 3.1 Glycolytic shift: the Warburg effect

A hallmark of hypoxia-induced metabolic adaptation in cancer cells is the preferential use of glycolysis over oxidative phosphorylation (OXPHOS), even when oxygen is sufficiently available—a phenomenon known as the Warburg effect (62). Originally described by Otto Warburg in the 1920s, this metabolic reprogramming allows cancer cells to meet their bioenergetic and biosynthetic demands through aerobic glycolysis, generating ATP rapidly and producing metabolic intermediates essential for cell proliferation (11). Hypoxia accentuates this shift, largely mediated by the stabilization and transcriptional activity of hypoxia-inducible factor-1α (HIF-1α), which serves as a central regulator of glycolytic metabolism under low oxygen conditions (59). Zhang et al. demonstrated that HIF-1 inhibits mitochondrial biogenesis and oxidative phosphorylation by antagonizing the activity of the transcription factor c-Myc. Specifically, HIF-1α suppresses c-Myc–dependent transactivation of genes critical for mitochondrial function, such as *PGC-1β* and *TFAM*, as well as those encoding components of the electron transport chain. This results in reduced mitochondrial mass and oxygen consumption, shifting the tumor cells toward a glycolytic phenotype even under normoxic conditions (63).

HIF-1α upregulates several genes involved in glucose uptake and glycolytic flux. Semenza et al. demonstrated that HIF-1 plays a central role in regulating the transcription of genes encoding glycolytic enzymes under low oxygen conditions. The authors show that HIF-1 binds to hypoxia response elements (HREs) in the promoters of several glycolytic genes, including *aldolase A*, *enolase 1*, and *lactate dehydrogenase A* (LDHA), leading to increased gene transcription (64). Notably, it induces the expression of glucose transporters, particularly GLUT1 (SLC2A1) and GLUT3 (SLC2A3), enhancing the influx of glucose into hypoxic tumor cells (65, 66). Chen et al. demonstrated that hypoxia induces *GLUT1* mRNA expression in a HIF-1α-dependent manner in multiple tumor cell lines. Using promoter-reporter assays, they identified a functional HRE within the *GLUT1* promoter that mediates transcriptional activation by HIF-1α. Moreover, they show that oncogenic H-Ras enhances *GLUT1* induction under hypoxia by increasing HIF-1α protein levels (67). Concurrently, HIF-1α activates key glycolytic enzymes, including hexokinase 2 (HK2), which catalyzes the phosphorylation of glucose to glucose-6-phosphate, and pyruvate kinase M2 (PKM2), which governs the final step of glycolysis by converting phosphoenolpyruvate to pyruvate (68). These enzymatic modifications boost glycolytic throughput, favoring the rapid production of ATP under anaerobic conditions. Riddle et al. demonstrated that exposure to hypoxic conditions significantly increases HK2 mRNA and protein levels, suggesting a transcriptional regulatory mechanism. Promoter analysis and deletion studies indicate the presence of a HRE in the HK2 promoter region, implicating HIF-1 as a key transcription factor mediating this response (69). Papandreou et al. demonstrated that, under hypoxic conditions HIF-1α directly induces transcription of pyruvate dehydrogenase kinase 1 (PDK1), which phosphorylates and inactivates the pyruvate dehydrogenase (PDH) complex. By blocking PDH activity, pyruvate is prevented from entering the TCA cycle, reducing mitochondrial oxygen consumption and favoring glycolysis (70).

Rather than being shuttled into mitochondria for oxidative metabolism, pyruvate generated from glycolysis is predominantly reduced to lactate by lactate dehydrogenase A (LDHA)—another transcriptional target of HIF-1α. Semenza et al. showe that HIF-1 binds to HREs in the promoters of several glycolytic genes, including *lactate dehydrogenase A* (LDHA), leading to increased gene transcription (64). This step regenerates NAD^+^, ensuring the continuity of glycolysis (71). The accumulation of lactate within the cell is managed by the upregulation of monocarboxylate transporters (MCTs), particularly MCT4, which facilitates lactate efflux into the extracellular space (72). This export system prevents intracellular acidification but contributes to the development of an acidic TME.

The implications of this metabolic shift are multifaceted. Firstly, it allows cancer cells to survive and proliferate in poorly oxygenated niches by maintaining ATP production without reliance on oxygen-dependent mitochondrial respiration (11). Secondly, the acidification of the extracellular milieu supports tumor invasion and metastasis by promoting extracellular matrix degradation and facilitating epithelial-to-mesenchymal transition (EMT) (73, 74). Further, Gao et al. showed that, acidic extracellular microenvironment enhances the invasiveness of prostate cancer PC-3 cells by upregulating the secretion of cathepsin B, a protease involved in extracellular matrix degradation (75). Thirdly, lactate and the acidic pH act as potent modulators of the immune response, suppressing the activity of cytotoxic T lymphocytes and natural killer (NK) cells while favoring the recruitment of immunosuppressive regulatory T cells (Tregs) and TAMs. Moreover, lactate hampers dendritic cell maturation and antigen presentation, thereby weakening adaptive immune activation (75). However, some study also demonstrate that, lactate increases stemness of CD8+ T cells to enhance anti-tumor immunity (76). This contrasting role of lactate on CD8+ activity underscores the necessity for further investigation to determine the specific conditions within the tumor microenvironment under which lactate either inhibits or enhances immune responses. Thus, the glycolytic phenotype not only fuels tumor growth but also fosters an immunoevasive and pro-metastatic microenvironment.

### 3.2 Mitochondrial metabolism and redox homeostasis

While the glycolytic shift under hypoxia is a defining feature of tumor metabolism, alterations in mitochondrial function and redox homeostasis are equally critical to cancer cell adaptation (77). Under low oxygen conditions, HIF-1α acts as a key modulator of mitochondrial metabolism, actively repressing oxidative phosphorylation (OXPHOS) to reduce cellular oxygen consumption and mitochondrial-generated reactive oxygen species (ROS), which can be toxic at high levels (78). One of the primary mechanisms by which HIF-1α exerts this control is through the induction of pyruvate dehydrogenase kinase 1 (PDK1), a mitochondrial enzyme that phosphorylates and inactivates pyruvate dehydrogenase (PDH). Kim et al. demonstrated that HIF-1α induces pyruvate dehydrogenase kinase 1 (PDK1), which inhibits pyruvate dehydrogenase activity and redirects pyruvate away from the mitochondria (79). This action prevents the conversion of pyruvate into acetyl-CoA, thereby reducing the entry of carbon substrates into the tricarboxylic acid (TCA) cycle and downstream OXPHOS activity.

By inhibiting PDH activity, HIF-1α effectively shifts metabolic flux away from the mitochondria and reinforces the glycolytic phenotype (79). This not only minimizes mitochondrial oxygen consumption under hypoxia but also limits the generation of mitochondrial ROS—a byproduct of electron leakage from the electron transport chain (ETC) (80). However, hypoxia itself paradoxically increases mitochondrial ROS production, especially at complex III of the ETC. This moderate ROS generation acts as a signaling molecule rather than a source of oxidative damage (81, 82). Notably, ROS serve to stabilize HIF-1α under hypoxic conditions by inhibiting prolyl hydroxylases, further enhancing HIF-1α activity and establishing a positive feedback loop that sustains the hypoxia response.

In addition to regulating enzymatic flux, HIF-1α also plays a role in suppressing mitochondrial biogenesis. It downregulates transcriptional coactivators such as PGC-1α, a master regulator of mitochondrial biosynthesis, and modulates the expression of mitochondrial DNA-encoded components of the ETC (78, 83). Scharping et al. found that, the TME induces metabolic insufficiency in intratumoral CD8^+^ T cells by suppressing mitochondrial biogenesis. This repression occurs through sustained antigen stimulation and inhibitory signaling, leading to downregulation of PGC-1α, a master regulator of mitochondrial metabolism. As a result, T cells within tumors exhibit diminished mitochondrial mass, membrane potential, and respiratory capacity, contributing to their functional exhaustion. The study further showed that restoring mitochondrial biogenesis through PGC-1α overexpression enhances T cell metabolic fitness and anti-tumor function, highlighting the critical role of metabolic regulation in determining T cell efficacy in the TME (84). This reduction in mitochondrial mass is an adaptive response that helps cancer cells limit oxidative damage and maintain redox balance under chronic hypoxic stress.

Moreover, hypoxic tumor cells reprogram their antioxidant systems to cope with elevated ROS levels. Enzymes such as superoxide dismutase (SOD), glutathione peroxidase (GPx), and peroxiredoxins (PRDXs) are often upregulated to neutralize ROS and preserve redox homeostasis (85). Maintaining this delicate balance is crucial for cancer cell survival, as excessive ROS can trigger mitochondrial dysfunction, DNA damage, and apoptosis. Conversely, a controlled increase in ROS promotes oncogenic signaling, angiogenesis, and therapy resistance (86).

### 3.3 Glutamine metabolism

In addition to glucose, glutamine serves as a critical metabolic substrate for cancer cells, especially under hypoxic conditions where oxidative metabolism is suppressed (87). Leone et al. show that blocking glutamine metabolism in tumor cells not only inhibits their growth but also reprograms the tumor microenvironment to enhance anti-tumor immunity. Glutamine blockade impairs tumor cell metabolism while simultaneously promoting oxidative metabolism and effector function in CD8^+^ T cells (88). Glutamine metabolism, or glutaminolysis, plays a vital role in maintaining biosynthetic capacity, redox homeostasis, and mitochondrial function suppressed (87, 89). Tumor cells exhibit a high demand for glutamine, utilizing it as both a carbon and nitrogen source to support survival and proliferation (89). Under hypoxia, when the flux through the tricarboxylic acid (TCA) cycle is attenuated due to HIF-1α–mediated inhibition of pyruvate dehydrogenase (via PDK1), glutamine becomes increasingly important for anaplerosis—the replenishment of TCA cycle intermediates necessary for the synthesis of macromolecules (79, 90).

A key adaptation of hypoxic cancer cells is the reductive carboxylation of glutamine-derived α-ketoglutarate (α-KG) to produce citrate, which is then used for lipid biosynthesis (91, 92). This reaction is catalyzed in a reverse direction by isocitrate dehydrogenase (IDH1/2), primarily in the cytosol or mitochondria, and becomes prominent when oxidative metabolism is constrained. This pathway not only supports membrane lipid synthesis essential for cell proliferation but also generates NADPH, a crucial reducing equivalent that fuels antioxidant defenses and anabolic reactions (91, 92). Thus, under hypoxia, glutamine metabolism contributes significantly to maintaining redox balance by ensuring a continuous supply of NADPH, which in turn supports the regeneration of reduced glutathione (GSH) and other antioxidant systems (93).

Glutamine also serves as a nitrogen donor for the *de novo* synthesis of non-essential amino acids, nucleotides, and hexosamines—critical components for rapidly dividing tumor cells (94, 95). Moreover, the carbon skeleton of glutamine helps sustain mitochondrial function by feeding into alternative TCA cycle routes, thereby supporting mitochondrial bioenergetics despite the downregulation of canonical OXPHOS (96). In many hypoxic tumors, mitochondrial respiration is selectively preserved or reprogrammed to adapt to the dual pressures of low oxygen and high proliferative demand, and glutamine metabolism contributes to this fine-tuned metabolic plasticity (10, 97).

Although HIF-1α does not directly regulate the transcription of glutaminolysis-related genes to the same extent as glycolytic genes, indirect modulation through signaling pathways influenced by HIFs has been reported (8, 98). Wise et al. demonstrated that, MYC upregulates glutaminase (GLS) —the enzyme that converts glutamine to glutamate—, and MYC is frequently upregulated downstream of HIF signaling, suggesting an indirect route by which hypoxia can modulate glutamine metabolism (99). Likewise, HIF-driven alterations in mitochondrial dynamics and oxygen sensing may indirectly influence the activity of IDH enzymes, promoting the switch toward reductive glutamine metabolism (100, 101).

Importantly, glutamine metabolism also contributes to tumor-induced immunosuppression. Tumor cells can outcompete infiltrating T cells for glutamine, thereby depriving effector immune cells of this essential nutrient (102). Edwards et al. demonstrated that the glutaminase inhibitor CB-839 (telaglenastat) enhances T-cell antitumor activity in triple-negative breast cancer (TNBC) models by blocking tumor glutamine metabolism. This inhibition reduces cancer cell proliferation while sparing T cells, which adapt via oxidative metabolism, leading to improved infiltration and reduced immunosuppression in the tumor microenvironment (102). This metabolic competition impairs T cell proliferation, cytokine production, and cytotoxic function.

In addition, glutamine supports the differentiation and suppressive activity of Tregs and myeloid-derived suppressor cells (MDSCs), further tipping the balance toward an immunosuppressive TME (103, 104). Oh et al. showed that targeting glutamine metabolism with a glutamine antagonist disrupts the differentiation and suppressive function of myeloid-derived suppressor cells (MDSCs) and Tregs within the tumor microenvironment (105). Furthermore, TAMs can utilize glutamine to drive an M2-like polarization state, which promotes tumor growth and immune evasion (11). Liu et al. revealed that TAMs utilize glutamine metabolism to drive an M2-like, immunosuppressive phenotype that supports tumor growth. Specifically, glutamine is converted into α-ketoglutarate (α-KG), a key metabolite that orchestrates M2 polarization through both metabolic and epigenetic mechanisms. α-KG promotes the activity of the histone demethylase Jmjd3, leading to demethylation of repressive histone marks and induction of M2-associated gene expression (106). Glutamine-derived metabolites such as α-KG and NADPH may also modulate epigenetic and redox-dependent pathways that reinforce the tolerogenic phenotype of immune cells within the TME (87, 107). Thus, glutamine metabolism not only sustains tumor bioenergetics and biosynthesis but also shapes an immunosuppressive niche that facilitates cancer progression (107).

### 3.4 Lipid metabolism

In addition to reprogramming glucose and glutamine metabolism, hypoxia significantly influences lipid metabolic pathways in cancer cells (108, 109). Lipids are essential not only as energy reservoirs but also as building blocks for membrane biosynthesis and signaling molecules (110). Under hypoxic conditions, HIF-1α orchestrates a metabolic shift that favors lipid uptake, synthesis, and storage while modulating fatty acid oxidation in a context-dependent manner (77, 111). Seo et al. demonstrated that fatty acids stimulate the expression of fatty acid-binding protein 5 (FABP5), which in turn stabilizes and activates hypoxia-inducible factor-1 (HIF-1) in liver cancer cells. This FABP5/HIF-1 axis reprograms lipid metabolism by upregulating genes involved in lipid uptake and synthesis, thereby enhancing cancer cell proliferation (112).

One of the key adaptations observed is the upregulation of lipid biosynthetic enzymes under hypoxia. HIF-1α has been shown to enhance the expression of fatty acid synthase (FASN), which catalyzes the synthesis of long-chain fatty acids from acetyl-CoA and malonyl-CoA (113). Additionally, hypoxia stimulates the expression of lipid transport proteins, such as CD36 and FABP3, facilitating the increased uptake of extracellular fatty acids which aids in tumor proliferation. Chabowski et al. found that exposure to hypoxic conditions significantly increases the translocation of fatty acid transporters, particularly FAT/CD36 and FABPpm, from intracellular compartments to the sarcolemma in rat hearts (114). Nath et al. demonstrated that elevated uptake of free fatty acids through the lipid transporter CD36 promotes epithelial-mesenchymal transition (EMT) in hepatocellular carcinoma (HCC) cells (115). FABP3 and FABP7 are transcriptionally upregulated by hypoxia via HIF-1α, increasing lipid uptake and supporting tumor proliferation (116). Bensaad et al. demonstrated that under hypoxic conditions, lipid droplet (LD) accumulation results primarily from FABP3/7-mediated fatty acid uptake, while *de novo* lipogenesis is downregulated. Additionally, during hypoxia-reoxygenation, the source of ATP generation varies by cell type, relying either on β-oxidation of fatty acids or glycogen breakdown to support cellular energy needs (116). Cordero et al. identified FABP7 as a critical metabolic regulator in HER2-positive breast cancer brain metastases. They showed that FABP7 promotes tumor cell survival and growth within the brain microenvironment by enhancing fatty acid uptake, lipid storage, and oxidative metabolism (117). This surge in lipid influx and synthesis contributes to the accumulation of lipid droplets (LDs)—cytoplasmic organelles that store neutral lipids such as triglycerides and cholesteryl esters (116, 118).

Lipid droplets serve multiple adaptive functions in hypoxic tumor cells. First, they act as energy reserves, supplying fatty acids that can be mobilized through β-oxidation when glucose availability is limited (116). Second, lipid droplets help buffer reactive oxygen species (ROS) by sequestering excess fatty acids that might otherwise undergo peroxidation and cause cellular damage. In doing so, they contribute to the maintenance of redox homeostasis, a crucial factor for cancer cell survival under stress (119, 120). Moreover, certain lipid types stored within LDs, such as polyunsaturated fatty acids (PUFAs), can be mobilized for the synthesis of lipid signaling mediators involved in inflammation, angiogenesis, and immune modulation (110, 120, 121).

However, the role of fatty acid oxidation (FAO) in hypoxic tumors is more nuanced. In many cancer types, FAO is downregulated under hypoxia to reduce mitochondrial oxygen consumption and minimize ROS production, a strategy that aligns with the broader suppression of oxidative metabolism. This is often achieved through HIF-1α–mediated transcriptional repression of key enzymes involved in β-oxidation, such as carnitine palmitoyltransferase 1A (CPT1A). Du et al. showed that HIFs drive lipid accumulation and tumorigenesis in clear cell renal cell carcinoma (ccRCC) by repressing key enzymes of fatty acid metabolism. Specifically, HIF suppressed carnitine palmitoyltransferase 1A (CPT1A) and medium-chain acyl-CoA dehydrogenase (MCAD), leading to impaired fatty acid β-oxidation and enhanced lipid droplet deposition (122). Huang et al. demonstrated that HIF-1α downregulates medium- and long-chain acyl-CoA dehydrogenases (MCAD and LCAD), key enzymes in the β-oxidation pathway, through a PGC-1β–mediated mechanism. This suppression decreased FAO, lowered ROS production, and supported cancer cell proliferation (113). In gastric adenocarcinoma tissues, Ezzeddini et al, noted that elevated HIF-1α levels were accompanied by reduced expression of FAO genes, including CPT1A, LCAD, MCAD, and PPARγ. Clinical data also showed that low CPT1A and PPARγ correlated with poor prognosis (114, 123). Conversely, in some tumor contexts—such as prostate cancer or certain subsets of breast cancer—FAO may be maintained or even upregulated to provide ATP and NADPH under metabolic stress, suggesting that lipid metabolism is highly plastic and tumor-type specific. For instance, Jariwala et al. reported that fatty acid β-oxidation and the enzyme CPT1A play a critical role in the growth and survival of hormone receptor-positive (HR+) breast cancer cells. Their findings revealed that CPT1A, a pivotal regulator of fatty acid metabolism, is overexpressed in HR+ tumors and is necessary for promoting cell proliferation, survival, and the formation of colonies and mammospheres (124). Simmilarly, in ErbB2^+^ breast cancer models, Nandi et al. noted that the enzyme CPT1A is essential for tumor growth, angiogenesis, and metastasis. CPT1A ablation reduced ATP and NADPH, disrupted mitochondrial function, and increased oxidative stress—revealing that FAO serves as a key source of metabolic energy in these tumors (125). Using castration-resistant prostate cancer (CRPC) models, Joshi et al. showed that CPT1A overexpression enhances FAO, increasing ATP production, acyl-carnitine levels, serine biosynthesis, and ROS defense. FAO supported cell proliferation—even in androgen-deprived conditions—and higher CPT1A was associated with poor progression-free survival (126).

## 4 Hypoxia and metabolic crosstalk in the tumor microenvironment

### 4.1 Interactions with cancer-associated fibroblasts

Hypoxia plays a central role in orchestrating metabolic interactions between cancer cells and stromal components, particularly cancer-associated fibroblasts (CAFs), which are among the most abundant and metabolically active cell types in the TME (1, 127). Under hypoxic stress, cancer cells not only adapt their own metabolism but also actively reprogram neighboring CAFs, establishing a metabolic symbiosis that facilitates tumor growth, survival, and resistance to therapy (1). One of the key outcomes of this metabolic crosstalk is the induction of the “reverse Warburg effect, “ wherein CAFs adopt a glycolytic phenotype even in the presence of oxygen and supply energy-rich metabolites to support oxidative metabolism in adjacent cancer cells (1, 128).

In hypoxic tumors, soluble factors such as transforming growth factor-beta (TGF-β), interleukin-6 (IL-6), and hypoxia-inducible factors (particularly HIF-1α) trigger the activation and metabolic reprogramming of fibroblasts (129–132). These activated CAFs upregulate glycolytic enzymes, glucose transporters (GLUT1, GLUT3), and lactate dehydrogenase A (LDHA), leading to enhanced conversion of glucose to lactate and ketone bodies (1). Unlike the traditional Warburg effect observed in cancer cells, which use glycolysis for self-sustained growth, the reverse Warburg effect reflects a cooperative strategy, wherein CAFs undergo aerobic glycolysis and secrete metabolic intermediates into the extracellular space. These high-energy metabolites are then taken up by cancer cells and funneled into the tricarboxylic acid (TCA) cycle and oxidative phosphorylation (OXPHOS) pathways to fuel biosynthesis and ATP production (128, 133).

The lactate and ketones exported by CAFs are transported via monocarboxylate transporters (MCTs)—particularly MCT4 in CAFs for export and MCT1 in cancer cells for import—forming a bidirectional shuttle system (134, 135). This lactate shuttle not only fuels mitochondrial metabolism in cancer cells but also contributes to acidification of the extracellular environment, which promotes invasion, immune evasion, and angiogenesis (131, 136). Lactate itself functions as a potent immunomodulatory metabolite: it suppresses the cytotoxic function of CD8+ T cells and natural killer (NK) cells, inhibits dendritic cell maturation, and enhances the activity of Tregs, all of which contribute to the establishment of an immunosuppressive TME (137). Brand et al. demonstrated that elevated lactic acid production, driven by lactate dehydrogenase A (LDHA) in tumors, suppresses antitumor immune responses by impairing the function of cytotoxic T lymphocytes (CTLs) and natural killer (NK) cells (138). Gottfried et al. showed that tumor-derived lactic acid impairs dendritic cell function by inhibiting their activation and reducing the expression of co-stimulatory molecules and pro-inflammatory cytokines. This metabolic byproduct also downregulates antigen-presenting capacity, ultimately compromising the ability of DCs to initiate effective T-cell responses (139). Colegio et al. revealed that tumor-derived lactic acid plays a crucial role in functionally polarizing macrophages toward an M2-like, immunosuppressive phenotype. The study demonstrated that lactic acid produced by glycolytically active tumor cells induces the expression of arginase 1 and vascular endothelial growth factor (VEGF) in macrophages via HIF-1α stabilization, promoting tissue remodeling and angiogenesis while dampening anti-tumor immunity (140). Watson et al. showed that lactic acid in the TME sustains regulatory T cells (Tregs) by promoting lactate uptake via monocarboxylate transporter 1 (MCT1). Unlike effector T cells, Tregs metabolically adapt to lactic acid, enhancing their stability and immunosuppressive function in the TME (141).

Additionally, CAFs directly participate in immune modulation by secreting immunosuppressive cytokines such as TGF-β CXCL12, and IL-6, which promote the expansion of immunosuppressive cell populations, including Tregs and myeloid-derived suppressor cells (MDSCs), and suppress CD8+ T cells, natural killer (NK) cells, and dendritic cells (DCs) (142–144). These cytokines also downregulate antigen presentation and impair the recruitment and activation of effector T cells (144). In some contexts, CAFs upregulate immune checkpoint ligands such as PD-L1, further dampening T cell–mediated anti-tumor responses. Costa et al. using single-cell transcriptomic profiling, identified distinct CAF subpopulations, including inflammatory CAFs (iCAFs) and myofibroblastic CAFs (myCAFs), each with unique gene expression signatures and functional roles. Notably, iCAFs were enriched in immune-regulatory genes and were shown to express immune checkpoint molecules including PD-L1, contributing to T-cell dysfunction and immune exclusion (145). The dense extracellular matrix (ECM) produced by CAFs under hypoxic and inflammatory signals also serves as a physical barrier to immune cell infiltration and can sequester growth factors and chemokines in a manner that favors tumor immune evasion (146, 147).

Interestingly, this metabolic and immunological coupling offers a survival advantage to cancer cells, particularly in heterogeneous oxygen landscapes, where some regions remain normoxic while others are severely hypoxic. In normoxic tumor zones, cancer cells can utilize oxidative metabolism more efficiently by scavenging CAF-derived metabolites, thereby conserving their own glucose and glutamine pools (148). Meanwhile, in hypoxic niches, lactate recycling allows cells to bypass the limitations of impaired mitochondrial function (148). The resulting immunosuppressive milieu, reinforced by both metabolic byproducts and cytokine signaling, ensures not only metabolic flexibility but also immune escape, promoting tumor progression and therapeutic resistance.

### 4.2 Immune cell modulation

Hypoxia not only reprograms the metabolism of cancer cells and stromal fibroblasts but also exerts a profound influence on the immune landscape of the TME. Through both direct effects on immune cell metabolism and indirect modulation of signaling pathways, hypoxia facilitates immune evasion and establishes an immunosuppressive milieu that favors tumor survival and progression (3, 149). The accumulation of metabolic byproducts—such as lactate—and the activation of hypoxia-inducible transcriptional programs are central to this immunomodulatory process.

One of the most prominent features of hypoxia-induced metabolic reprogramming is the excessive production of lactate, resulting from enhanced glycolysis in both cancer cells and stromal components like cancer-associated fibroblasts (11, 131). Lactate accumulation leads to acidification of the extracellular environment, which impairs the function and viability of cytotoxic immune effector cells, particularly CD8+ T cells and natural killer (NK) cells. These immune cells require oxidative metabolism for proliferation and effector function; however, in the hypoxic, lactate-rich TME, they face metabolic competition, nutrient deprivation, and redox stress, leading to exhaustion, reduced cytokine production, and impaired cytolytic activity (11, 131). Fischer et al. in their study observed that, lactic acid inhibited the proliferation and cytokine production (such as IFN-γ and IL-2) of human cytotoxic and helper T cells in a dose-dependent manner. Additionally, lactic acid impaired T cell motility and cytolytic activity. These effects were not due to acidity alone but specifically linked to the lactate anion (150). Brand et al. demonstrated that lactic acid production driven by lactate dehydrogenase A (LDHA) in tumor cells plays a pivotal role in suppressing tumor immunosurveillance by both T cells and natural killer (NK) cells. Lactic acid accumulation inhibited cytokine production (e.g., IFN-γ, TNF-α), cytolytic activity, and proliferation of CD8^+^ T cells and NK cells. Additionally, lactic acid reduced the expression of key effector molecules and altered immune cell metabolism, further dampening anti-tumor responses (138). Husain et al. noted that lactate impaired NK cell cytotoxicity and interferon-gamma (IFN-γ) production, thereby weakening their ability to target and kill tumor cells (151). Also activities of dendritic cells are impaired. Gottfried et al., demonstrated that lactic acid produced by tumor cells significantly impairs dendritic cell (DC) function. Exposure to tumor-derived lactic acid suppressed the expression of co-stimulatory molecules (CD80, CD86) and MHC class II on DCs, reducing their ability to activate T cells (139). In parallel, high lactate concentrations promote the differentiation of Tregs and myeloid-derived suppressor cells (MDSCs), further dampening anti-tumor immunity (152). Angelin et al. demonstrated that Foxp3, the master transcription factor of Tregs, reprograms their metabolism to thrive in low-glucose, high-lactate environments. The study revealed that Foxp3 suppresses glycolysis and promotes oxidative metabolism, enabling Tregs to utilize lactate as an energy source (153). Rao et al, demonstrated that acidic conditions, rather than lactate itself, play a pivotal role in promoting the differentiation of FOXP3^+^ Tregs from conventional CD4^+^ T cells. Using *in vitro* and murine tumor models, they showed that lactic acid-induced acidity enhances TGF-β–mediated Treg induction through a pH-dependent mechanism. This process involves the lactylation of moesin, a cytoskeletal protein that stabilizes TGF-β receptor signaling (154). In their commentary, Tuomela and Levings elucidated that acidic conditions—specifically lactic acid–driven low pH—not the lactate ion itself—potently augment the TGF-β–mediated differentiation of conventional CD4^+^ T cells into FOXP3^+^ Tregs. They highlight that tumor-generated lactic acid acidifies the microenvironment, which, in the presence of TGF-β, fosters Treg development via enhanced lactylation of proteins like moesin that bolster TGF-β receptor signaling (155). Also, Husain et al. found that elevated levels of tumor-derived lactate enhanced the accumulation and suppressive function of MDSCs (151). Similarly, Yang et al., revealed that lactate accumulation in the TME enhances the immunosuppressive function of MDSCs, contributing to radioresistance in pancreatic cancer. Mechanistically, lactate activated the HIF-1α/GPR81/mTOR/STAT3 signaling pathway in MDSCs, promoting their expansion and suppressive capacity (156).

In addition, HIF-1α signaling contributes directly to immune evasion mechanisms by upregulating immune checkpoint molecules. Notably, HIF-1α induces the transcription of programmed death-ligand 1 (PD-L1) on tumor cells and immune cells such as dendritic cells and macrophages. Noman et al. identified PD-L1 as a direct transcriptional target of HIF-1α in the context of hypoxia. They demonstrated that HIF-1α binds to a hypoxia response element (HRE) within the PD-L1 promoter, leading to its upregulation on both tumor cells and myeloid-derived suppressor cells (MDSCs) (157). Ding et al. found that hypoxia significantly upregulated PD-L1 expression in glioma cells in a HIF-1α-dependent manner. Chromatin immunoprecipitation (ChIP)- qPCR analysis confirmed that HIF-1α directly binds to the PD-L1 proximal promoter region, enhancing its transcription (158). Engagement of PD-L1 with its receptor PD-1 on T cells delivers an inhibitory signal that curtails T cell activation, proliferation, and cytotoxicity (159). This immune checkpoint axis becomes especially active in hypoxic niches, where HIF-driven PD-L1 expression reinforces T-cell anergy and tolerance, allowing tumor cells to escape immune surveillance (160). This mechanism is clinically significant, as tumors with high HIF-1α and PD-L1 expression are often resistant to immunotherapies unless checkpoint inhibitors are used in combination with agents that normalize the TME (160).

Another key immune population affected by hypoxia is TAMs. Hypoxic conditions skew macrophage polarization toward an M2-like phenotype, characterized by anti-inflammatory, tissue-remodeling, and pro-tumorigenic functions. Leblond et al., demonstrated that hypoxia induces the polarization and re-education of macrophages toward an M2-like phenotype in glioblastoma models (U87 and U251). Under hypoxic conditions, macrophages exhibited increased expression of M2 markers (such as CD206) and decreased pro-inflammatory cytokine production. Notably, even macrophages initially activated to an M1 phenotype could be reprogrammed into an M2-like state when exposed to hypoxia (161). Zhang et al., found that tumor hypoxia enhances metastasis in non-small cell lung cancer (NSCLC) by selectively promoting the polarization of macrophages toward an M2 phenotype. This process was mediated through the activation of the ERK signaling pathway. Under hypoxic conditions, tumor cells induced M2-associated markers (such as CD206 and IL-10) in macrophages (162). M2 macrophages thrive in hypoxic zones due to their metabolic reliance on glycolysis and their capacity to adapt to low-oxygen environments (163). These M2-polarized TAMs secrete vascular endothelial growth factor (VEGF), arginase-1, interleukin-10 (IL-10), and transforming growth factor-beta (TGF-β), which collectively support angiogenesis, suppress adaptive immunity, and facilitate tumor invasion and metastasis (11). Furthermore, HIF-2α in macrophages enhances the expression of genes involved in immune suppression and angiogenesis, consolidating the pro-tumoral role of TAMs in hypoxic regions (164).

### 4.3 Angiogenesis and vascular remodeling

A hallmark of the hypoxic TME is the induction of angiogenesis, a process critical for supplying oxygen and nutrients to rapidly proliferating tumor cells (165). Hypoxia, through stabilization of hypoxia-inducible factors (HIFs)—especially HIF-1α—serves as a master regulator of this process (166). In response to low oxygen tension, HIF-1α activates the transcription of a broad array of pro-angiogenic genes, most notably vascular endothelial growth factor A (VEGF-A) (167). VEGF-A promotes the proliferation, migration, and survival of endothelial cells, leading to the sprouting of new blood vessels from pre-existing vasculature (168). Alongside VEGF, HIF-1α also induces other angiogenic mediators such as angiopoietins, platelet-derived growth factor (PDGF), and stromal-derived factor-1 (SDF-1), which collectively orchestrate the complex remodeling of the tumor vasculature (166, 169).

However, the angiogenesis induced by hypoxia in tumors is typically aberrant and disorganized, resulting in leaky, tortuous, and poorly functional blood vessels (152). These newly formed vessels are often structurally immature, lacking pericyte coverage and proper basement membrane integrity. As a result, they fail to provide efficient perfusion, leading to heterogeneous oxygen distribution within the tumor mass (19, 170). Paradoxically, while angiogenesis is initiated to alleviate hypoxia, the resulting vasculature frequently fails to resolve oxygen deficiency, thereby creating a vicious cycle of persistent hypoxia and continued HIF activation (19, 165). This self-reinforcing loop drives further metabolic adaptation, immune suppression, and tumor aggressiveness.

In addition to fueling neovascularization, hypoxia-induced metabolic changes also impact the phenotype and function of endothelial cells (171, 172). Endothelial cells in the hypoxic TME undergo glycolytic reprogramming, increasing their reliance on anaerobic glycolysis for ATP production. This glycolytic reliance is largely driven by hypoxia-inducible factor 1-alpha (HIF-1α) by upregulating key glycolytic enzymes such as GLUT1, PFKFB3, LDHA, and PDK1. Studies show that ECs produce approximately 80–85% of their ATP through glycolysis, supporting critical processes such as and tip-cell formation during angiogenic sprouting, migration, and proliferation. PFKFB3, in particular, acts as a important metabolic regulator, and its inhibition has been shown to reduce pathological angiogenesis, normalize tumor vasculature, and suppress metastasis (173). Rohlenova et al., by employing single-cell RNA sequencing investigated the metabolic heterogeneity of endothelial cells (ECs) during pathological angiogenesis. The researchers mapped distinct metabolic states of ECs, revealing profound plasticity in glycolysis, oxidative phosphorylation, and fatty acid metabolism. They identified novel endothelial subpopulations with unique metabolic profiles and uncovered that metabolic reprogramming is tightly linked to specific angiogenic phenotypes. Importantly, targeting key metabolic pathways, such as PFKFB3-mediated glycolysis, selectively impaired pathological vessel formation without affecting normal vasculature, highlighting the therapeutic potential of metabolic interventions (174). Xu et al. revealed that hypoxia induces the overexpression of prolyl 4-hydroxylase subunit alpha 1 (P4HA1), which plays a key role in promoting post-ischemic angiogenesis. Mechanistically, P4HA1 enhances glycolytic activity in endothelial cells by down regulating fructose-1, 6-bisphosphatase 1 (FBP1), a negative regulator of glycolysis. This metabolic shift supports endothelial cell proliferation and neovascularization under hypoxic conditions. Tumor-derived lactate can further enhance glycolytic activity in ECs (175). Sonveaux et al. demonstrated that lactate acts as a signaling molecule in endothelial cells by inducing HIF-1α activation and promoting tumor angiogenesis. This effect is mediated through monocarboxylate transporter 1 (MCT1), which facilitates lactate uptake into endothelial cells (176). Furthermore, lactate produced by glycolytic tumor and stromal cells acts as a signaling molecule that stimulates VEGF expression and enhances endothelial cell motility and tube formation, thereby linking metabolic waste accumulation to vascular remodeling (177). This glycolytic phenotype is a hallmark of tumor-associated endothelial cells and represents a potential therapeutic target to disrupt tumor angiogenesis and restore vascular normalization (178).

The consequences of aberrant angiogenesis extend beyond oxygen supply. Leaky vessels contribute to interstitial fluid pressure (IFP), impair drug delivery, and allow for tumor cell intravasation, thereby facilitating metastasis (179–181). Boucher et al. investigated IFP in both tissue-isolated and subcutaneous tumors using human colon carcinoma (LS174T) and murine mammary carcinoma (MCaIV) models. They found that avascular tumors exhibited near-zero IFP, while vascularized tumors displayed significantly elevated and uniformly high IFP throughout their core, with steep pressure gradients only at the tumor periphery. These findings highlighted the role of aberrant tumor angiogenesis in generating interstitial hypertension due to leaky, immature blood vessels and impaired lymphatic drainage. The study provided critical insight into how elevated IFP can act as a barrier to the delivery of therapeutic agents, emphasizing the need to normalize tumor vasculature to improve treatment efficacy (182). Moreover, regions of poor perfusion become hypoxic and acidic, exerting selective pressure on tumor cells to adopt more aggressive and therapy-resistant phenotypes (181, 183). Attempts to normalize the tumor vasculature, such as with anti-VEGF therapies, have shown limited success, often due to the complex feedback loops involving hypoxia, metabolism, and angiogenic signaling (170).

## 5 Clinical implications and therapeutic targeting

Tumor hypoxia and metabolic reprogramming have profound clinical implications, as they drive tumor aggressiveness, metastasis, immune evasion, and resistance to chemotherapy, radiotherapy, and targeted therapies. Hypoxia-inducible factors (HIFs) orchestrate adaptive transcriptional programs that enhance glycolysis, angiogenesis, acidosis, and survival signaling, while metabolic rewiring toward aerobic glycolysis, glutaminolysis, and lipid metabolism supports rapid proliferation and therapeutic resistance. Clinically, elevated expression of HIF-1α, CAIX, GLUT1, LDHA, and lactate transporters correlates with poor prognosis across multiple malignancies. These insights have led to therapeutic strategies targeting hypoxia and tumor metabolism, including HIF-2α inhibitors (e.g., belzutifan), hypoxia-activated prodrugs, anti-angiogenic agents, glycolysis and glutaminase inhibitors, lactate transport blockers, CAIX inhibitors, and immunometabolic modulators aimed at reversing hypoxia-induced immune suppression [**Table 1**]. Combination approaches integrating metabolic inhibitors with chemotherapy, radiotherapy, or immunotherapy are emerging as promising strategies to overcome resistance and improve clinical outcomes.

**Table 1:** Therapeutic strategies targeting hypoxia and metabolic reprogramming in cancer.

Therapeutic strategy	Target/Pathway	Mechanism of action	Examples of agents/Approaches	Clinical status/Applications	Advantages	Limitations/Challenges
HIF Inhibitors	HIF-1α/HIF-2α	Inhibit stabilization, dimerization, or transcriptional activity of HIFs	PT2385, PT2977 (Belzutifan), Acriflavine	Belzutifan FDA-approved (VHL-associated RCC); others in trials	Directly blocks hypoxia-driven gene programs	Redundancy between HIF isoforms; resistance mechanisms
Prolyl Hydroxylase (PHD) Modulation	PHD-HIF axis	Alters HIF degradation pathway	Experimental inhibitors/activators	Preclinical/early clinical	Modulates oxygen-sensing machinery	Systemic effects on normal tissues
Hypoxia-Activated Prodrugs (HAPs)	Hypoxic tumor regions	Selectively activated in low oxygen conditions	Tirapazamine, Evofosfamide (TH-302)	Clinical trials	Tumor-selective cytotoxicity	Variable hypoxia levels; limited phase III success
Anti-Angiogenic Therapy	VEGF/VEGFR	Blocks tumor neovascularization induced by hypoxia	Bevacizumab, Sunitinib, Sorafenib	Approved in multiple cancers	Starves tumor of oxygen and nutrients	Induces adaptive hypoxia and resistance
Glycolysis Inhibitors	HK2, PFKFB3, LDHA	Suppress Warburg effect and lactate production	2-Deoxy-D-glucose, FX11, 3PO	Preclinical/early trials	Targets metabolic addiction of tumors	Toxicity to normal proliferating cells
Lactate Transport Inhibitors	MCT1/MCT4	Blocks lactate efflux and metabolic symbiosis	AZD3965	Phase I trials	Disrupts tumor-stroma metabolic coupling	Tumor metabolic flexibility
Glutaminase Inhibitors	GLS1	Inhibits glutamine metabolism	CB-839 (Telaglenastat)	Clinical trials	Effective in glutamine-dependent tumors	Compensatory metabolic pathways
mTOR Inhibitors	PI3K/AKT/mTOR	Reduces metabolic signaling and HIF translation	Everolimus, Temsirolimus	Approved in RCC, breast cancer	Dual metabolic and growth inhibition	Feedback activation of pathways
AMPK Activators/Metformin	AMPK-mTOR axis	Inhibits anabolic metabolism; improves metabolic stress response	Metformin	Clinical trials/repurposed drug	Safe, cost-effective	Variable efficacy in non-diabetics
Fatty Acid Oxidation (FAO) Inhibitors	Carnitine palmitoyl transferase 1 CPT1	Blocks lipid metabolism	Etomoxir	Preclinical	Targets lipid-dependent tumors	Hepatotoxicity concerns
ROS-Modulating Agents	Oxidative stress pathways	Exploits hypoxia-induced ROS imbalance	Buthionine sulfoximine (BSO)	Experimental	Enhances sensitivity to chemo/radiation	Redox adaptation
Carbonic Anhydrase IX (CAIX) Inhibitors	pH regulation	Disrupts extracellular acidification	SLC-0111	Clinical trials	Targets hypoxia-specific pH adaptation	Tumor heterogeneity
Immunometabolic Targeting	Adenosine pathway, IDO, lactate	Reverses hypoxia-induced immune suppression	IDO inhibitors, CD39/CD73 inhibitors	Clinical trials	Enhances immunotherapy response	Complex immune-metabolic interactions
Combination Radiotherapy with Hypoxia Modifiers	Oxygenation status	Enhances radiation sensitivity	Hyperbaric oxygen, Nimorazole	Clinical use in some cancers	Improves radiosensitivity	Limited accessibility

### 5.1 Targeting HIF pathways

Given the central role of hypoxia-inducible factors (HIFs) in orchestrating a wide array of tumor adaptations—including survival under hypoxic stress, neovascularization, immune evasion, and metabolic reprogramming—targeting HIF signaling has emerged as a promising therapeutic strategy in oncology (98, 184, 185). Among the HIF family members, HIF-1α and HIF-2α have been most extensively studied due to their distinct but overlapping roles in cancer biology. HIF-1α primarily regulates glycolytic metabolism and acute hypoxic responses, while HIF-2α is more involved in chronic hypoxia, tumor proliferation, and stemness. Notably, in malignancies such as ccRCC, often driven by von Hippel–Lindau (VHL) gene mutations—constitutive stabilization of HIF-2α is a critical driver of tumor progression (186).

Therapeutic targeting of HIF pathways offers the unique opportunity to simultaneously disrupt multiple cancer hallmarks, including abnormal glucose metabolism (e.g., Warburg effect), pathological angiogenesis via VEGF induction, and resistance to apoptosis and immune surveillance (98, 184, 185). This multi-pronged disruption can be particularly advantageous in tumors heavily reliant on the hypoxic microenvironment for survival and growth.

One of the most significant advancements in this field is the development of PT2385, a first-in-class, orally bioavailable, selective HIF-2α inhibitor. PT2385 binds to the PAS-B domain of HIF-2α, a region crucial for heterodimerization with HIF-1β (ARNT), effectively preventing DNA binding and transcriptional activation of downstream oncogenic targets such as VEGF, cyclin D1, and GLUT1 (187). Preclinical studies have demonstrated that PT2385 leads to marked tumor regression in VHL-deficient RCC, supporting its selective efficacy (187). Using genetically engineered mouse models and patient-derived xenografts (PDXs), Chen et al. showed that PT2385 selectively disrupts the heterodimerization of HIF-2α with ARNT, thereby inhibiting the transcription of downstream oncogenic targets such as VEGF, EPO, and cyclin D1. Treatment with PT2385 resulted in significant tumor regression and prolonged survival in preclinical models of VHL-deficient ccRCC, without notable systemic toxicity (188). Phase I dose-escalation clinical trial, revealed that PT2385 was well tolerated, with no dose-limiting toxicities. Pharmacodynamic analyses confirmed target engagement through suppression of HIF-2α–regulated gene expression (189). In patients with first recurrence of glioblastoma, PT2385 was evaluated in a Phase II single-arm clinical trial (NCT03216499), where the median progression-free survival (PFS) was 1.8 months and the median overall survival (OS) was 7.7 months. Importantly, exploratory analyses indicated that higher systemic exposure to PT2385 and increased tumor acidity (as measured by CEST MRI) were associated with longer PFS, suggesting a potential pharmacodynamic effect (190). Building on these results, PT2977 (MK-6482 or belzutifan), a second-generation HIF-2α antagonist with improved potency and pharmacokinetics, has shown promising activity in clinical trials. Phase II studies in advanced ccRCC have reported durable disease control with a favorable safety profile (191, 192), FDA in 2021 approved belzutifan for patients with VHL-associated RCC (193). The FDA granted approval based on the clinically significant improvement in overall response rate (ORR) demonstrated in patients participating in study MK-6482-004 (NCT03401788) and later for advanced sporadic clear-cell RCC that has progressed through multiple treatments in 2023 (194). Its ongoing evaluation in phase III trials, including combinations with immune checkpoint inhibitors (e.g., pembrolizumab) [LITESPARK-012], underscores the potential of HIF-2α inhibition as a cornerstone in the treatment of hypoxia-driven tumors (195).

Beyond direct inhibition, indirect targeting of the HIF pathway is also being actively pursued (98). Prolyl hydroxylase domain (PHD) enzymes, which regulate HIF stability through oxygen-dependent hydroxylation and subsequent VHL-mediated proteasomal degradation, are a focus of therapeutic interest. Chintala et al. (2012) reported that selenium suppresses tumor growth in ccRCC (VHL mutated ccRCC cells RC2) by inducing degradation of HIF-1α and HIF-2α through a prolyl hydroxylase 2 (PHD2)-dependent but VHL-independent pathway. Given that accumulation of HIF-α proteins drives oncogenesis in VHL-deficient ccRCC, this finding identifies selenium as a potential therapeutic agent capable of targeting hypoxia signaling even in the absence of functional VHL (196). Gastelum et al. showed that restoration of the oxygen-sensing function of PHD3 regulates HIF-2α expression and enhances the sensitivity of myeloma cells to hypoxia-induced apoptosis. Using human myeloma cell lines obtained from the American Type Culture Collection (ATCC), the study demonstrated that PHD3 restoration, by using a lentivirus vector or overcoming PHD3 epigenetic silencing using a demethyltransferase inhibitor, 5-Aza-2’-deoxycytidine (5-Aza-dC), suppresses hypoxia-driven HIF-2α stabilization, thereby promoting apoptotic cell death (197). Choi et al. investigated the effects of KRH102053 (2-amino-4-methylsulphanyl-butylic acid-4-methoxy-6-(4-methoxy-benzylamino)-2, 2-dimethyl-chroman-3-yl ester), a novel small-molecule activator of PHD2, in human HOS osteosarcoma, rat PC12 phaeochromocytoma and human HepG2 hepatoma cells. They demonstrated that KRH102053 significantly accelerates the degradation of HIF-1α by enhancing PHD2 activity, even under hypoxic conditions where HIF-1α is typically stabilized. This degradation leads to the suppression of HIF-1α target genes involved in angiogenesis and survival, such as VEGF (198).

Other indirect approaches include targeting upstream regulators like the PI3K/Akt/mTOR pathway, which enhances HIF-1α translation. Agents such as rapamycin (sirolimus) and temsirolimus have demonstrated partial HIF suppression and anti-angiogenic effects in various tumors. Lang et al. (2005) demonstrated that inhibition of mTOR signaling by rapamycin effectively suppresses HIF-1α activity in human gastric cancer cells, thereby impairing hypoxia-driven angiogenesis. *In vitro* experiments revealed reduced HIF-1α expression and transcriptional function following rapamycin treatment, while *in vivo* models showed significant inhibition of tumor vascularization and growth (199). Hu et al. investigated the anticancer efficacy of sirolimus (rapamycin), a known mTOR inhibitor, across various HNSCC cell lines and *in vivo* models. Their results demonstrated that sirolimus effectively suppressed tumor growth by inhibiting the mTOR signaling pathway, which plays a critical role in regulating cell proliferation, angiogenesis, and survival (200).

Furthermore, epigenetic modulators like histone deacetylase inhibitors (HDACi) (e.g., vorinostat/SAHA, panobinostat, and entinostat) and Bromodomain and Extraterminal (BET) inhibitors may suppress HIF transcription, while RNA interference (RNAi), CRISPR-mediated gene editing, and antisense oligonucleotides offer gene-specific silencing of HIF isoforms or their co-activators. HDACi reduce HIF output by blocking HIF transactivation and/or lowering HIF-1α protein (often via translational control) (201–203), while BET inhibitors (e.g., JQ1, OTX015/MK-8628, molibresib, pelabresib) limit recruitment of BRD proteins to hypoxia-responsive promoters, diminishing expression of HIF target genes like CA9 and VEGFA (204, 205). Using a combination of biochemical and functional assays. Fath et al., showed that HDACis reduce HIF-dependent gene expression by interfering with coactivator recruitment and transcriptional complex assembly, rather than by altering HIF-α acetylation status (206). Hutt et al. (2014) reported that the histone deacetylase inhibitor (HDACi) vorinostat suppresses HIF-1α expression by blocking its translation rather than affecting transcription or protein stability. Using cellular models (liver cancer-derived), the study showed that vorinostat reduces HIF-1α protein levels under hypoxic conditions, leading to impaired hypoxia-responsive gene expression (203). Further, Zhang et al. found that vorinostat significantly suppresses HIF-1α signaling by inhibiting its nuclear translocation, rather than altering its expression levels. This suppression was mediated through modulation of the importin α/β pathway and acetylation of Hsp90, a chaperone essential for HIF-1α stability and function (207). Although direct studies specifically linking BET inhibitors to HIF suppression are still emerging, existing literature acknowledges that BET bromodomain inhibition can suppress transcriptional programs driven by HIF and other oncogenic transcription factors (208). da Motta et al. showed that the BET bromodomain inhibitor JQ1 selectively disrupts hypoxia responses in triple-negative breast cancer (TNBC) using TNBC cell lines (including MDA-MB-231) and xenograft models. JQ1 treatment suppressed the expression of hypoxia-inducible genes, particularly carbonic anhydrase IX (CA9), and impaired angiogenesis, leading to reduced tumor adaptation under hypoxic conditions (205). Jenke et al. (2021) reviewed the therapeutic potential of HDACis in cancer. Despite promising preclinical data and several FDA-approved HDACis, their clinical efficacy as monotherapies has been limited by resistance and toxicity. The authors highlighted mechanism-based combination strategies—such as pairing HDACis with DNA-damaging agents, kinase inhibitors, immune checkpoint inhibitors, or proteasome inhibitors—that can enhance antitumor responses and overcome resistance (209). Several HDACis, such as vorinostat, panobinostat, and entinostat, have been tested either as single agents or in combination with chemotherapy and targeted therapies. While monotherapy trials showed limited efficacy, combinations—particularly with immune checkpoint inhibitors, PARP inhibitors, and cytotoxic drugs—demonstrated more promising results in improving therapeutic response (209, 210).

Hänze et al. investigated the effects of RNAi targeting HIF-1α in human lung epithelial cells (A549 cell line). They demonstrated that small interfering RNA (siRNA) specific to HIF-1α effectively reduced its mRNA and protein levels, leading to significant down-regulation of HIF-1α target genes, including vascular endothelial growth factor (VEGF). This inhibition of HIF-1α signaling resulted in a marked decrease in cell proliferation under hypoxic conditions (211). Gillespie et al. explored the therapeutic potential of silencing HIF-1α using RNAi in U251, U87, and U373 glioma cells. By employing short hairpin RNA (shRNA) to stably knock down HIF-1α expression, they observed significant reductions in the growth of glioma xenografts in immunodeficient mice (212). Xu et al., using RNA interference to suppress HIF-1α expression, demonstrated significant inhibition of tumor growth both *in vitro* and *in vivo*. The knockdown of HIF-1α led to increased apoptosis, as evidenced by elevated caspase-3 activity and upregulation of pro-apoptotic markers, along with downregulation of anti-apoptotic proteins (213). In hepatobiliary and pancreatic cancer cell lines (MIA PaCa-2), expression of HIF-1α-targeted siRNA vectors reduced HIF-1α mRNA (~13% of control) and protein (~41% of control) levels, blocking tumor growth *in vitro* and in nude-mouse xenografts (214). Liu et al. demonstrated that CRISPR/Cas9-mediated knockout of HIF-1α significantly enhances the antitumor efficacy of transarterial embolization (TAE) in hepatocellular carcinoma (HCC). The targeted deletion of HIF-1α in HCC cells resulted in reduced tumor growth, increased apoptosis, and suppressed angiogenesis in both *in vitro* and *in vivo* models (215). Greenberger et al. investigated the therapeutic potential of EZN-2968, a locked nucleic acid (LNA) antisense oligonucleotide designed to target HIF-1α mRNA. The study demonstrated that EZN-2968 effectively reduced HIF-1α mRNA and protein levels across multiple tumor cell lines under both normoxic and hypoxic conditions. This inhibition led to downregulation of HIF-1α target genes involved in angiogenesis, glucose metabolism, and cell survival (216).

Despite these advances, several challenges complicate the clinical translation of HIF-targeted therapies (98, 217). HIFs are intracellular transcription factors, long considered “undruggable” due to the absence of well-defined enzymatic pockets and the need to disrupt protein-protein or protein-DNA interactions (218, 219). Moreover, functional redundancy between HIF isoforms and compensatory upregulation of alternative survival pathways may limit the effectiveness of monotherapies (220, 221). There is also a risk of off-target effects and context-specific responses, especially in tissues with physiological hypoxia, such as the bone marrow and kidney (222, 223).

To overcome these limitations, combination strategies are under active exploration. Co-administration of HIF inhibitors with immune checkpoint blockade (e.g., anti-PD-1/PD-L1), angiogenesis inhibitors (e.g., bevacizumab), or metabolic modulators (e.g., glutaminase inhibitors) may produce synergistic antitumor responses. Preclinical data suggest that disrupting HIF-driven immunosuppression may enhance T cell infiltration and function within the TME, potentially improving responses to immunotherapy (224). Luo et al. demonstrated that elevated HIF-1α expression contributes to an immunosuppressive microenvironment by upregulating PD-L1 expression and impairing cytotoxic T cell function in NSCLC cell lines and mouse models. Through pharmacological and genetic inhibition of HIF-1α, they observed enhanced infiltration and activity of CD8+ T cells and improved antitumor responses when combined with anti-PD-1 therapy (225). Shurin and Umansky reviewed data showing HIF-1 inhibition boosted the complete response rate to anti-PD-1 from ~25% to ~67% in murine tumors by decreasing immunosuppressive TAMs and MDSCs while increasing CTLs and NK cells (224). Salman et al. explored the therapeutic potential of the novel HIF inhibitor 32-134D in a murine model of hepatocellular carcinoma (HCC). The study revealed that 32-134D effectively suppressed HIF-1α and HIF-2α activity, leading to reduced tumor angiogenesis, glycolysis, and immune evasion. When combined with anti-PD-1 immune checkpoint therapy, 32-134D significantly enhanced antitumor immunity, resulting in complete tumor eradication in a majority of treated mice. This combination therapy also promoted durable immune memory, preventing tumor recurrence (226). Falchook et al. conducted a Phase I clinical trial to evaluate bortezomib, a proteasome inhibitor that downregulates HIF-1α, with bevacizumab, an antiangiogenic agent, in patients with advanced solid tumors. The rationale was to simultaneously inhibit HIF-1α signaling and VEGF-mediated angiogenesis, two key pathways driving tumor growth under hypoxic conditions. The combination was generally well tolerated, with manageable side effects, and showed signs of antitumor activity, including disease stabilization in a subset of patients (227). Nishii et al. investigated a novel triple therapy combining osimertinib (a third-generation EGFR inhibitor), bevacizumab (an anti-VEGF antibody), and cetuximab (an anti-EGFR antibody) in EGFR-mutant non-small cell lung cancer (NSCLC) characterized by high expression of HIF-1α and TGF-α. The study aimed to overcome resistance mechanisms driven by hypoxia and compensatory EGFR signaling. The combination therapy showed promising antitumor efficacy, particularly in models with elevated HIF-1α and TGF-α levels, suggesting that simultaneous inhibition of EGFR, VEGF, and hypoxia-related pathways can synergistically suppress tumor progression and may represent a viable strategy for treating resistant EGFR-mutant NSCLC (228).

### 5.2 Targeting metabolic enzymes

Tumor cells in a hypoxic microenvironment undergo profound metabolic reprogramming to meet their bioenergetic and biosynthetic demands under low oxygen tension (229). This reprogramming creates a unique metabolic dependency that renders cancer cells vulnerable to selective metabolic enzyme inhibitors (16). Therapeutic targeting of these enzymes offers a promising strategy to disrupt cancer cell survival mechanisms without affecting normal cells, which have more metabolic flexibility (16). Several key metabolic pathways altered by hypoxia—such as glycolysis, glutaminolysis, and lipid metabolism—have emerged as viable therapeutic targets, with multiple compounds under preclinical and clinical investigation.

*Lactate dehydrogenase A (LDHA)* is a critical enzyme that converts pyruvate to lactate, regenerating NAD^+^ to sustain anaerobic glycolysis. Overexpressed in many tumors, particularly under hypoxic conditions, LDHA supports both energy production and acidification of the TME (230). Cui et al. demonstrated that LDHA is overexpressed in pancreatic cancer tissues and upregulated under hypoxia via HIF-1/2α binding to its promoter. This HIF-dependent LDHA induction was associated with increased glycolytic activity and lactate production, supporting metabolic adaptation of pancreatic cancer cells to hypoxic TME. Using gene knockdown and overexpression approaches, they further showed that silencing HIF-1α or HIF-2α significantly reduced hypoxia-induced LDHA expression (231). Sheng et al. found that LDHA expression was significantly elevated in HCC cell line (HCCLM3 cell), correlating with advanced tumor stage and poor patient prognosis. Using RNA interference (RNAi), mediated by lentiviral vectors, to knock down LDHA in HCC cells, the authors observed marked reductions in cell proliferation, colony formation, migration, and invasion, along with decreased lactate production and glycolytic activity. *In vivo*, LDHA silencing suppressed tumor growth and lung metastasis in mouse xenograft models. These findings suggest that LDHA promotes HCC progression through its role in aerobic glycolysis and highlight it as a potential metabolic target for anti-cancer therapy (232). LDHA inhibitor such as FX11 have shown efficacy in preclinical models by impairing tumor growth and enhancing reactive oxygen species accumulation (233). Rellinger et al. investigated the effects of FX11, on neuroblastoma cell lines. FX11 treatment significantly reduced aerobic glycolysis, as evidenced by decreased lactate production, and suppressed cell proliferation *in vitro*. Furthermore, FX11 impaired tumor growth in a murine xenograft model, highlighting its potential as a metabolic-targeted therapy (234). Gao et al. demonstrated that both pharmacological inhibition using FX11 and genetic silencing of LDHA effectively suppressed tumor progression in osteosarcoma cell lines. LDHA inhibition led to reduced lactate production, impaired aerobic glycolysis, decreased cell proliferation, and increased apoptosis *in vitro*, as well as significant tumor growth suppression *in vivo* (235). However, clinical development has been limited due to toxicity and bioavailability challenges (236, 237).

*Pyruvate dehydrogenase kinase 1 (PDK1)* is another attractive target due to its role in blocking the entry of pyruvate into the tricarboxylic acid (TCA) cycle, thereby suppressing mitochondrial oxidative phosphorylation. Under hypoxia, HIF-1α upregulates PDK1, reinforcing the glycolytic phenotype (238). The small molecule dichloroacetate (DCA) inhibits PDK1, reactivating mitochondrial metabolism and inducing apoptosis in cancer cells by reversing the Warburg effect. Bonnet et al. identified that cancer cells exhibit suppression of the mitochondria–potassium (mitoK^+^) channel axis, which is crucial for maintaining mitochondrial function and apoptosis regulation. Pharmacological activation or genetic restoration of this axis, using agents such as DCA to inhibit pyruvate dehydrogenase kinase, reactivated mitochondrial oxidative phosphorylation, increased reactive oxygen species production, and triggered apoptosis in cancer cells. In preclinical models, normalization of mitoK^+^ channel function significantly reduced tumor growth without harming normal cells, suggesting this metabolic–mitochondrial pathway as a promising therapeutic target for cancer treatment (239). Wong et al. reported that DCA, shifts endometrial cancer cell metabolism from glycolysis to mitochondrial oxidative phosphorylation, leading to mitochondrial depolarization, increased reactive oxygen species production, and induction of apoptosis. DCA treatment significantly reduced cell viability and enhanced caspase activation *in vitro*, supporting its potential as a metabolic modulator with therapeutic promise in endometrial cancer (240). Madhok et al. demonstrated that DCA promotes metabolic shift from glycolysis to mitochondrial oxidative phosphorylation in colorectal cancer cells. This metabolic reprogramming led to mitochondrial membrane depolarization, increased reactive oxygen species generation, induction of apoptosis, and cell-cycle arrest at the G_2_ phase. DCA treatment significantly reduced cell proliferation *in vitro*, highlighting its potential as a metabolic-targeted therapeutic approach for colorectal cancer (241). Michelakis et al. showed that DCA inhibits pyruvate dehydrogenase kinase in glioblastoma, shifting metabolism from glycolysis to mitochondrial oxidative phosphorylation. In animal models and a small cohort of glioblastoma patients, DCA reduced tumor growth and showed evidence of clinical benefit, supporting its potential as a metabolic therapy for glioblastoma (242). Dunbar et al. conducted a Phase I dose-escalation trial of oral DCA in adults with recurrent malignant brain tumors to assess safety, tolerability, and pharmacodynamics. The study established a maximum tolerated dose (MTD) based on the development of dose-limiting peripheral neuropathy. DCA treatment showed measurable pharmacodynamic effects, including metabolic modulation, but clinical efficacy was limited, with no significant tumor regressions observed (243). While DCA has shown modest activity in clinical trials, its repurposing potential and low cost make it a candidate for combination therapies. Li et al. reported that DCA and metformin synergistically inhibited ovarian cancer cell growth by targeting complementary metabolic pathways. The combination treatment enhanced mitochondrial oxidative phosphorylation, reduced glycolysis, and increased reactive oxygen species production, leading to mitochondrial membrane depolarization, activation of apoptosis, and suppression of cell proliferation. *In vivo* experiment in nude mice, co-administration of DCA and metformin significantly reduced tumor growth compared to either agent alone (244). Olszewski et al. evaluated the *in vitro* cytotoxic effects of combining DCA with platinum-based chemotherapeutic agents in various cancer cell lines. The study found that DCA enhanced the anticancer activity of cisplatin and oxaliplatin, leading to greater reductions in cell viability compared to either agent alone (245). Haugrud et al. demonstrated that DCA enhances the cytotoxic effects of metformin in breast cancer cell lines by promoting oxidative damage and apoptosis. Combined treatment with DCA and metformin significantly increased ROS generation, disrupted mitochondrial membrane potential, and activated caspase-dependent cell death pathways, while also reducing lactate production, indicating suppression of aerobic glycolysis. These findings suggest that metabolic modulation with DCA can potentiate the anticancer efficacy of metformin through synergistic disruption of cancer cell energy metabolism (246). Powell et al. conducted a Phase II trial evaluating DCA, in combination with standard chemoradiotherapy for unresected, locally advanced head and neck squamous cell carcinoma (HNSCC). The regimen was generally well tolerated, with manageable toxicities, and demonstrated expected pharmacodynamic effects consistent with metabolic modulation. While the addition of DCA did not produce a statistically significant improvement in clinical outcomes compared to historical controls, the study confirmed the feasibility of integrating metabolic-targeting agents with conventional therapy in HNSCC and provided a rationale for further investigation in selected patient populations (247).

*Glutaminase (GLS)*, the enzyme responsible for converting glutamine to glutamate, is a key player in glutaminolysis, supporting anaplerosis and redox balance in hypoxic tumors (248). CB-839 (Telaglenastat) is a potent and selective GLS inhibitor that has progressed to clinical trials for various solid tumors and hematologic malignancies (**Table 2**) (249–253). By depleting glutamate pools and reducing the supply of biosynthetic intermediates, CB-839 impairs tumor growth, particularly in tumors dependent on glutamine metabolism. Clinical trials of glutaminase inhibitors (**Tables 2**), particularly telaglenastat (CB-839), have shown that targeting cancer metabolism via GLS inhibition is feasible and generally well tolerated, with consistent evidence of on-target activity. In early-phase studies, telaglenastat demonstrated partial metabolic responses and modest antitumor signals across solid tumors. In renal cell carcinoma (RCC), the ENTRATA trial reported improved progression-free survival (PFS) with telaglenastat plus everolimus compared with everolimus alone, though the difference did not reach conventional statistical significance. However, the larger CANTATA trial (telaglenastat + cabozantinib) failed to improve PFS over cabozantinib alone, tempering enthusiasm for its use in unselected RCC populations. Basket trials and immunotherapy combinations (e.g., with nivolumab) have shown acceptable safety but only limited or inconsistent efficacy signals. Other next-generation agents, such as IPN60090 (IACS-6274), have demonstrated early signs of clinical activity and effective GLS1 inhibition in Phase I studies, with ongoing trials exploring combinations (e.g., with bevacizumab and paclitaxel). Similarly, DRP-104 (sirpiglenastat), a glutamine antagonist that indirectly inhibits GLS-driven pathways, is in early-phase evaluation, showing manageable safety and rationale for combination with immunotherapy (atezolizumab) (249–253).

**Table 2:** Notable clinical trials of glutaminase (GLS/GLS1) inhibitors.

Drug (target)	Trial/NCT	Phase	Population/Setting	Regimen	Key notes/status (as of Aug 30, 2025)
Telaglenastat (CB-839; GLS1)	Phase I dose-escalation/expansion NCT02071862	I	Advanced/metastatic solid tumors	Telaglenastat (oral), various combos allowed in expansion	Established RP2D; showed on-target GLS inhibition and signals of antitumor activity; generally well tolerated (249).
Telaglenastat (CB-839; GLS1)	ENTRATA – NCT03163667	II	Heavily pretreated advanced RCC	Telaglenastat + everolimus vs placebo + everolimus	Improved median PFS 3.8 vs 1.9 mo (HR 0.64; one-sided P=0.079); tolerable (250).
Telaglenastat (CB-839; GLS1)	CANTATA – NCT03428217	II (randomized, dbl-blind)	Previously treated metastatic clear-cell RCC	Telaglenastat + cabozantinib a TKI vs placebo + cabozantinib	Did not improve PFS vs control; safety consistent with components (251).
Telaglenastat (CB-839; GLS1)	BEGIN basket – NCT03872427	II (basket)	Solid tumors with specific genomic alterations	Telaglenastat monotherapy	Ongoing/posted basket design evaluating mutation-selected cohorts.
Telaglenastat (CB-839; GLS1)	(multi-cohort) Study CX-839-004; NCT02771626)	I/II	Metastatic melanoma, RCC, others	Telaglenastat + nivolumab	Generally well tolerated; no consistent efficacy signal across cohorts (252).
Telaglenastat (CB-839; GLS1)	NCT03965845	I/II	patients with solid tumors	Telaglenastat + CDK4/6 inhibitor palbociclib	Ongoing
IPN60090 (IACS-6274; GLS1)	First-in-human [NCT03894540.]	I	Molecularly selected advanced cancers	IPN60090 (oral)	Early antitumor activity; effective GLS1 inhibition; acceptable safety (253).
IPN60090 (IACS-6274; GLS1)	NCT05039801	I	Advanced solid tumors	IPN60090 ± bevacizumab & paclitaxel	Ongoing dose-finding/combination study to define MTD/RP2D and safety.
DRP-104 (sirpiglenastat; glutamine antagonist affecting GLS pathway)	NCT04471415	I/IIa	Advanced solid tumors	DRP-104 IV or SC, as monotherapy and with atezolizumab	Characterizing safety/PK/PD and preliminary antitumor activity; routes being compared; combination arm with atezolizumab.

MTD, Maximum Tolerated Dose; RP2D, Recommended Phase 2 Dose; TKI, tyrosine kinase inhibitor.

*Lipid metabolism* is also a critical axis in hypoxia-adapted tumors. HIF signaling enhances lipid uptake and storage, while tumor cells regulate fatty acid synthesis and oxidation to maintain energy and redox balance (111, 254). Fatty acid synthase (FASN), and carnitine palmitoyltransferase 1 (CPT1), key enzymes in lipogenesis and fatty acid oxidation (FAO), respectively, are being explored to disrupt this balance. Targeting FASN can deprive cancer cells of membrane biosynthesis substrates, while CPT1 inhibition blocks mitochondrial fatty acid entry, thereby reducing ATP production in FAO-dependent tumors. *Ventura* and colleagues demonstrated that blocking the *de novo* synthesis of palmitate by inhibiting FASN with TVB-3166 triggers apoptosis in xenograft tumor growth. The treatment disrupted lipid raft integrity in cell membranes, thereby perturbing critical downstream signaling pathways—including PI3K–AKT–mTOR and Wnt–β-catenin—and reprogramming the expression of oncogenic genes such as c-Myc. These effects were tumor-cell specific: TVB-3166 suppressed anchorage-independent growth under lipid-rich culture conditions and significantly inhibited xenograft tumor growth *in vivo*, at concentrations aligned with its biochemical IC_50_ for FASN and palmitate synthesis (255). Li and colleagues uncovered a compelling mechanism by which FASN contributes to sorafenib resistance in hepatocellular carcinoma (HCC): FASN binds to HIF-1α, preventing its ubiquitination and degradation, thereby enhancing HIF-1α’s nuclear translocation and driving the transcriptional upregulation of SLC7A11—an anti-ferroptosis gene. This chain of events curtails sorafenib-induced ferroptosis, undermining the drug’s efficacy. Importantly, pharmacologically inhibiting FASN with orlistat restores ferroptotic sensitivity and synergistically enhances antitumor effects when combined with sorafenib across both *in vitro* and *in vivo* models, suggesting that targeting the FASN/HIF-1α/SLC7A11 axis may overcome sorafenib resistance in HCC (256). FASN inhibitors show strong preclinical antitumor activity, disrupting lipid metabolism and oncogenic signaling while inducing apoptosis. TVB-2640 is the only compound to progress into Phase I/II clinical trials, showing early efficacy signals but not yet regulatory approval. Other inhibitors (Fasnall, GSK2194069, IPI-9119, orlistat, TVB-3166, TVB-3664) remain at the preclinical or translational stage, limited by pharmacokinetic challenges, toxicity concerns, or lack of clinical advancement. Thus, while biologically compelling, FASN inhibition remains experimental, with future progress depending on biomarker-guided trials and rational drug combinations [**Table 3**] (255, 257–271). Cao et al. reported the discovery of a novel FASN inhibitor—termed 6p—derived from a focused library of platensimycin analogs. This compound selectively targets the ketosynthase (KS) domain of mammalian FASN and exhibits stronger cytotoxicity and higher selectivity compared to established inhibitors like orlistat and TVB-3166. *In vitro*, 6p demonstrated potent inhibitory effects against non-small cell lung cancer (NSCLC) (A549, NCI-H1299) and melanoma (A375) cells, while sparing normal cell lines. It induced G_2_/M cell cycle arrest, apoptosis, and significantly impaired colony formation, migration, and invasion. Importantly, in mouse xenograft models, 6p produced robust antitumor activity against NSCLC and melanoma, indicating its promise as a selective and effective FASN-targeted cancer therapy for future research and trials (260). In ccRCC, a hallmark is the accumulation of lipid- and glycogen-rich cytoplasmic droplets—a trait largely driven by loss of VHL function and subsequent stabilization of hypoxia-inducible factors (HIFs). Du et al. showed that HIF-1 and HIF-2 directly repress the gene encoding the mitochondrial fatty acid transporter CPT1A. This repression impairs fatty acid entry into mitochondria, reducing β-oxidation and diverting fatty acids into lipid droplets for storage. Restoring CPT1A expression curbs lipid accumulation and notably impairs tumor growth *in vivo*. Clinical data corroborated these findings, revealing lower CPT1A levels in human ccRCC compared to normal kidney tissue—an alteration associated with poorer patient outcomes (122). Bensaad et al. revealed that under hypoxic stress, HIF-1α drives the induction of fatty acid binding proteins FABP3 and FABP7, leading to lipid droplet accumulation through enhanced fatty acid uptake (rather than new lipid synthesis). This stored lipid pool is crucial: during reoxygenation, it supplies ATP (via β-oxidation or glycogen breakdown, depending on cell type) and shields cells from reactive oxygen species–induced damage. Disruption of lipid droplet formation—by knocking down FABP3, FABP7, or adipophilin—not only compromises cell survival after hypoxia–reoxygenation *in vitro* but also significantly impairs tumor growth *in vivo* (116).

**Table 3:** FASN inhibitors with their status and key insight.

Inhibitor	Type/Mechanism	Development stage	Key findings in cancer models	Clinical status/Notes
Fasnall	Small-molecule, covalent FASN inhibitor	Preclinical	Induces apoptosis, inhibits tumor growth, effective in breast cancer and glioblastoma xenografts	No clinical trials yet; remains experimental (257, 258).
GSK2194069	Potent, selective small-molecule FASN inhibitor	Preclinical	Suppresses *de novo* lipogenesis, induces metabolic stress, strong anti-proliferative effects in breast and prostate cancers	Development discontinued after early preclinical work; not advanced to clinic (258–260).
IPI-9119	Irreversible, covalent FASN inhibitor (targeting β-ketoacyl reductase domain)	Preclinical/early development	Shown to impair lipid biosynthesis, inhibit proliferation in multiple tumor cell lines, including hematologic malignancies	Not yet in clinical trials (261, 262).
Orlistat	FDA-approved anti-obesity drug, inhibits FASN thioesterase domain (off-target)	Repurposed (preclinical/early clinical exploration)	Inhibits tumor growth, induces apoptosis, modulates lipid metabolism in prostate, breast, and colon cancers	Poor bioavailability and pharmacokinetics limit oncology use; not approved for cancer (263–266)
TVB-2640 (Denifanstat)	First-in-class, oral, selective FASN inhibitor	Phase I/II clinical trials	Demonstrated target engagement and reduced lipogenesis; antitumor activity in KRAS-mutant NSCLC, HER2+ breast, and ovarian cancers	Advanced to Phase II; tested in combination with paclitaxel, bevacizumab, and immunotherapy; still investigational (267–269).
TVB-3166	Orally bioavailable, reversible FASN inhibitor	Preclinical	Induces apoptosis, disrupts lipid raft integrity, inhibits PI3K–AKT–mTOR and β-catenin pathways, suppresses xenograft tumor growth	No clinical trials yet; foundational in establishing FASN as a therapeutic target (255, 270).
TVB-3664	Optimized next-generation analog of TVB-3166	Preclinical/translational studies	Higher potency, stronger pharmacologic activity, improved metabolic inhibition compared to TVB-3166	Investigational; not yet in clinical trials (271).

Together, these strategies demonstrate the therapeutic potential of targeting cancer cell metabolism in the context of hypoxia, particularly when combined with immunotherapy or standard chemoradiation.

### 5.3 Hypoxia-activated prodrugs

Hypoxia-activated prodrugs (HAPs) represent a rational strategy to exploit the hypoxic nature of tumors by selectively delivering cytotoxic agents to oxygen-deprived regions while sparing normoxic healthy tissue (271, 272). These prodrugs are bioreductively activated in low-oxygen environments by cellular reductases, leading to the release of active metabolites that cause DNA damage, inhibit replication, or disrupt essential cellular machinery (**Table 4**) (273–289). The selective cytotoxicity of HAPs helps overcome one of the major barriers in cancer therapy—poor drug penetration and efficacy in hypoxic tumor cores (271, 272).

**Table 4:** Selected Hypoxia-Activated Prodrugs and Clinical Trials.

Agent and clinical trial ID	Mechanism of activation	Cancer type(s)	Trial phase	Monotherapy/Combination therapy	Status/Notes
Tirapazamine (TPZ) [TROG 98.02]	One-electron reduction under hypoxia	Locally advanced Head & neck cancer	Phase II	TPZ, CIS, and radiation vs. 5-FU/CIS and radiation.	TPZ did not significantly improve overall survival or locoregional control (273).
Tirapazamine (TPZ) [TROG 02.02, HeadSTART]	One-electron reduction under hypoxia	Advanced head & neck cancer	Phase III	TPZ combined with CIS and radiation	No evidence that the addition of TPZ improves OS (274).
Tirapazamine (TPZ) [NCT00020696]	One-electron reduction under hypoxia	Platinum-sensitive ovarian or primary peritoneal cancers.	Phase II	TPZ plus cisplatin	Definite activity, howerver toxicity, primarily non-hematologic, was substantial (275).
Tirapazamine (TPZ) [CATAPULT I]	One-electron reduction under hypoxia	NSCLC	Phase III	TPZ plus cisplatin	TPZ enhances the activity of cisplatin; mild to moderate adverse events (276).
Tirapazamine (TPZ) [NCT00262821; GOG study]	One-electron reduction under hypoxia	Advanced cervix cancer	Phase III	CIS and irradiation versus CIS and TPZ and irradiation	Not superior to CIS chemoradiotherapy, was tolerable (277).
Tirapazamine (TPZ) [SWOG S0003]	One-electron reduction under hypoxia	Advanced NSCLC	Phase III	Carboplatin + paclitaxel ± tirapazamine	While the TPZ arm showed increased toxicity without a clear OS, the study successfully identified OPN as a valuable biomarker to predict how well patients with advanced NSCLC would respond to chemotherapy (278).
Tirapazamine (TPZ) (S0004)	One-electron reduction under hypoxia	Limited-stage small cell lung cancer	Phase I	TPZ + Cisplatin/Etoposide and Radiotherapy	Overall toxicity profile acceptable; observed favorable survival (279).
Tirapazamine (TPZ) [NCT02174549]	One-electron reduction under hypoxia	Unresectable hepatocellular carcinoma	Phase I	TPZ + transarterial embolization (TAE)	Safe and tolerable with encouraging results (280).
PR-104 [NCT01037556.]	Activated by nitroreductases in hypoxia (Converted to alcohol PR-104A)	AML, ALL.	Phase I/II	Monotherapy	Shows promise; major toxicity (grade 3/4 adverse events) includes myelosuppression (anemia 62%, neutropenia 50%, thrombocytopenia 46%), febrile neutropenia (40%), infection (24%), and enterocolitis (14%) (281).
PR-104	Activated by nitroreductases in hypoxia (Converted to alcohol PR-104A)	Advanced solid tumours	Phase I	Previous, median two prior chemotherapy regimens	Weekly PR-104 was tolerated at doses up to 670 mg/m², with dose-limiting myelotoxicity, establishing 675 mg/m² as the recommended phase II dose (282).
Evofosfamide (EVO; TH-302) [TH CR-406/SARC021]	Hypoxia-activated prodrug of Br-IPM	Unresectable/metastatic soft-tissue sarcomas.	Phase II/III	Doxorubicin plus Evo versus doxorubicin	Addition of Evo to doxorubicin as first-line therapy did not improve overall survival (283).
Evofosfamide (EVO; TH-302) [MAESTRO; NCT01746979]	Hypoxia-activated prodrug of Br-IPM	Pancreatic ductal adenocarcinoma	Phase III	Evo/Gem vs Placebo (Pbo)/Gem	Primary endpoint was not met, though it showed modest gains in PFS and ORR with a manageable safety profile (284).
Banoxantrone (AQ4N); [NCT00394628]	Reduced to AQ4, a DNA-binding cytotoxin	Glioblastoma multiforme	Phase Ib/IIa	AQ4N in Combination With Radiation + Temozolomide	To Evaluate the Safety, Tolerability, and Efficacy, Current status: Unknown Status
Banoxantrone (AQ4N)	Reduced to AQ4, a DNA-binding cytotoxin	Advanced cancers.	Phase I	Monotherapy	Well tolerated when administered weekly on a 3-of-4-week schedule at 768 mg/m(2) (285).
NI-Pano (CH-03)	Hypoxia -activated prodrug of panobinostat	Solid tumors (preclinical to early clinical)	–	–	Compelling mechanistic and pharmacologic rationale for developing NI-Pano as a hypoxia-selective anticancer agent (286, 287).
TPZ derivatives	Various structural analogs of tirapazamine	Solid tumors (preclinical)	–	–	Efforts ongoing to improve selectivity and reduce systemic toxicity (288, 289)

CIS, cisplatin; NSCLC, Non-small-cell lung cancer; OS, overall survival; OPN, osteopontin; GOG, Gynecologic Oncology Group; PFS, progression free survival; ORR, Objective Response Rate; Gem, Gemcitabine.

One of the earliest and most extensively studied HAPs is tirapazamine (TPZ), a benzotriazine di-N-oxide. TPZ undergoes enzymatic one-electron reduction under low-oxygen conditions, producing highly reactive radical species that cause DNA strand breaks and cytotoxicity specifically in hypoxic regions. Preclinical studies demonstrated strong synergy with DNA-damaging agents such as cisplatin and radiation, reflecting its ability to eliminate the hypoxic cell population that drives therapeutic resistance (290). Clinical trials showed encouraging antitumor activity, but results were variable due to heterogeneity in tumor hypoxia among patients and dose-limiting toxicities (**Table 4**) (273–289). Tirapazamine (TPZ) derivatives represent a next-generation class of hypoxia-activated prodrugs designed to overcome the limitations of TPZ, such as systemic toxicity and suboptimal selectivity for hypoxic tumor regions. Structural analogs of TPZ, including SAN-1, SN30000 (also known as CEN-209), and others, have been developed to enhance hypoxia selectivity, improve pharmacokinetics, and increase therapeutic efficacy. Preclinical studies have demonstrated that these derivatives retain potent DNA-damaging activity under hypoxic conditions while exhibiting reduced off-target effects in normoxic tissues. Among them, SN30000 has advanced the furthest, showing superior tissue penetration and stronger synergy with radiotherapy and chemotherapy compared to TPZ in animal models. While most TPZ derivatives remain at the preclinical stage, their promising profiles have spurred interest in advancing them into clinical development as part of combination regimens aimed at targeting hypoxia-driven tumor resistance (288, 289).

Another prominent HAP is PR-104. It has demonstrated activity against leukemia and various solid tumors and is currently under clinical investigation. However, toxicity such as myelo-suppression remains a challenge. Patterson et al. investigated PR-104, focusing on its mechanism of action and preclinical antitumor activity. PR-104 is a phosphate ester prodrug rapidly converted *in vivo* to PR-104A, which undergoes enzymatic reduction under hypoxic conditions (and in some tumors via aldo-keto reductase 1C3) to yield potent cytotoxic nitrogen mustards that induce inter strand DNA cross-links, leading to cell death. Preclinical studies in multiple human tumor xenograft models showed that PR-104 exhibited strong antitumor activity, particularly against hypoxic tumors, with significant tumor growth delay and, in some cases, regression (291). Singleton et al. examined the formation of DNA cross-links in a panel of 9 human tumor cell lines, treated with PR-104A, the active form of the hypoxia-activated prodrug PR-104, and their relationship to hypoxia, bioreductive metabolism, and cytotoxicity. The study showed that PR-104A induced extensive inter-strand DNA cross-links under hypoxic conditions, with cytotoxic potency correlating strongly with the extent of hypoxia and enzymatic reduction, particularly via aldo-keto reductase 1C3 in normoxic settings. Tumor cell sensitivity varied based on hypoxic fraction and reductase expression, highlighting dual activation pathways—oxygen-dependent and oxygen-independent (292). Foehrenbacher et al. investigated the contribution of bystander effects to the antitumor activity of PR-104. Using multicellular tumor spheroids and xenograft models, they demonstrated that the cytotoxic metabolites generated from PR-104A in hypoxic tumor cells can diffuse into neighboring, less hypoxic or normoxic cells, causing significant secondary cell killing. Mathematical modeling and experimental data indicated that this bystander effect substantially enhances overall tumor cell kill, especially in tumors with heterogeneous oxygenation (293). Benito et al. demonstrated that both murine and human leukemia models exhibit pronounced hypoxia, particularly within the bone marrow microenvironment, as confirmed by hypoxia markers and imaging. Evaluating PR-104, they found it to be highly effective *in vitro* and *in vivo*, producing significant cytotoxicity against leukemia cells under hypoxic conditions and markedly prolonging survival in mouse models. PR-104’s activity was attributed to its selective activation in hypoxic leukemic niches, targeting areas often resistant to conventional chemotherapy (294). The Phase I/II trial by Konopleva et al. evaluated the hypoxia-activated prodrug PR-104 in relapsed/refractory AML and ALL, exploiting its activation in hypoxic bone marrow niches or via AKR1C3 to overcome chemoresistance. Among 31 heavily pretreated patients, the maximum tolerated dose was 3 g/m², with dose-limiting toxicities mainly prolonged myelosuppression, mucositis, and infections. Although overall responses were limited, some patients showed blast reductions and one achieved CRi, with biomarker analysis indicating that higher AKR1C3 expression correlated with activity. The study concluded that while PR-104 demonstrated biological proof-of-principle for targeting hypoxia and metabolic vulnerabilities in leukemia, its clinical use is constrained by toxicity, supporting the need for biomarker-guided selection and combination approaches rather than monotherapy (281). McKeage et al. conducted a Phase I trial assessing weekly intravenous administration of PR-104A, in patients with advanced solid tumors. Dose escalation identified dose-limiting toxicities—primarily thrombocytopenia and neutropenia—with the maximum tolerated dose established at 675 mg/m² weekly. PR-104 showed linear pharmacokinetics, rapid conversion to PR-104A, and evidence of antitumor activity, including partial responses and prolonged stable disease in some patients (282). PR-104 plus sorafenib was trialed in advanced hepatocellular carcinoma (HCC). The study was poorly tolerated, mainly due to thrombocytopenia and neutropenia, leading to study discontinuation (295).

Other HAPs under investigation include TH-302 (Evofosfamide), which releases the alkylating agent bromo-isophosphoramide mustard in hypoxic regions. Evofosfamide has been evaluated in combination with standard chemotherapy in pancreatic cancer, soft tissue sarcoma, and glioblastoma, showing modest benefit and tolerability. Both the SARC021 (in sarcoma patients) and MAESTRO (in pancreatic cancer patients), phase III trials showed that adding evofosfamide to standard chemotherapy (doxorubicin in soft-tissue sarcoma and gemcitabine in pancreatic cancer) failed to improve overall survival compared to chemotherapy alone (283, 284). Borad et al. conducted a randomized Phase II trial comparing gemcitabine plus TH-302 with gemcitabine alone in patients with advanced pancreatic cancer. The combination therapy significantly improved progression-free survival (5.6 months vs. 3.6 months) but did not produce a statistically significant improvement in overall survival (296).

Skwarska et al. (2021) reported the development of a novel hypoxia-activated KDAC (lysine deacetylase) inhibitor (NI-Pano; CH-03)), designed to selectively target hypoxic tumor regions. Preclinical studies demonstrated that the compound effectively inhibited KDAC activity under low-oxygen conditions, leading to DNA damage, cell-cycle arrest, and apoptosis in cancer cells. In xenograft tumor models, the inhibitor showed selective activation in hypoxic regions, resulting in significant tumor growth suppression with minimal systemic toxicity (286, 287). So there are compelling mechanistic and pharmacologic rationale for developing NI-Pano as a hypoxia-selective anticancer agent (286, 287).

AQ4N (Banoxantrone) is a bioreductive prodrug that, under hypoxic conditions, is converted to AQ4, a potent DNA-binding cytotoxin and topoisomerase II inhibitor (297). Preclinical studies have shown that AQ4N enhances the sensitivity of tumors to radiotherapy, offering a dose-modifying factor of approximately 2—with limited additional normal tissue toxicity when scheduled appropriately (298). AQ4N reached Phase I clinical trials in solid tumors and lymphomas. Studies showed that it is selectively activated in hypoxic tumor regions, well tolerated at doses up to ~768 mg/m² (weekly) or 1, 200 mg/m² (every 21 days), with main side effects being mild fatigue, nausea, and blue skin/urine discoloration. Some patients achieved disease stabilization and one lymphoma patient had a partial response (285, 299, 300). Despite promising tumor-selective activation and tolerability, Phase II trials were largely discontinued (except briefly in glioblastoma) due to strategic and financial reasons, and development was not pursued further (301, 302).

However, hypoxia-activated prodrugs hold promise particularly as part of nanocarrier systems to further enhance their selectivity and minimize off-target effects. Despite some clinical setbacks, continued development of HAPs with improved hypoxia specificity and reduced toxicity remains a vibrant area of cancer research. Xu and colleagues addressed the limited clinical impact of TPZ, they designed a novel class of urea-containing TPZ derivatives. These derivatives exhibited dramatically enhanced hypoxic cytotoxicity—showing between 9.5- and 30.9-fold greater potency compared to TPZ, while preserving hypoxia selectivity. Among them, TPZP stood out, delivering approximately 20-fold higher cytotoxicity under hypoxia with a similar selectivity ratio. To maximize delivery and minimize systemic exposure, the researchers formulated TPZP into fibrin-targeting nanoparticles (FT11-TPZP-NPs). When used in combination with a vascular disrupting agent (CA4-NPs) to boost tumor fibrin deposition and intensify hypoxia, this nanodrug achieved remarkable efficacy: a 98.1% inhibition rate in CT26 tumor models (initial volume ~480 mm³) and complete eradication of tumors in 4 out of 6 treated subjects after a single dose (288). Ajnai et al. engineered a gold nanoparticle-based delivery system for tirapazamine (TPZ) to enhance its targeting and cytotoxicity against hypoxic tumors. The gold nanoparticles improved TPZ’s stability, solubility, and tumor-specific accumulation, enabling controlled release in hypoxic microenvironments. *In vitro* and *in vivo* studies demonstrated that TPZ-loaded nanoparticles generated higher levels of reactive oxygen species, induced greater DNA damage, and achieved stronger tumor growth inhibition compared to free TPZ, while reducing systemic toxicity (303). Hao et al. developed a novel transarterial chemoembolization (TACE) strategy for treating hepatocellular carcinoma (HCC) that overcomes the limitations of conventional formulations. They engineered bovine serum albumin (BSA) nanoparticles loaded with TPZ, to enable sustained drug release. In a rabbit VX2 liver cancer model, TACE using these BSATPZ nanoparticles combined with Lipiodol (LP) significantly enhanced antitumor efficacy compared to standard TACE—markedly delaying tumor progression, reducing lung metastases, and more effectively reshaping the TME. Notably, the therapy induced immunogenic cell death, suggesting the potential involvement of immune system reactivation in the treatment response (304). Zhao and colleagues developed an innovative two-stage nanoengineering strategy, using a gadolinium–europium metal–organic framework (GdEuMOF) loaded with tirapazamine (TPZ), designed to amplify TPZ activation through exacerbated hypoxia in breast tumors during microwave hyperthermia. In the first stage, microwave (MW) irradiation triggers enhanced tumor hypoxia indirectly via thrombus formation and circulatory disruption. In the second stage, the GdEuMOF@TPZ nanoparticles actively deplete residual oxygen through chemical reactions under MW exposure, creating a more uniformly hypoxic environment. This dual-layered exacerbation of hypoxia significantly enhances TPZ activation and synergistically boosts the efficacy of microwave hyperthermia–chemotherapy against breast cancer (305). Wang et al. developed a TH-302-loaded nanodrug to target hypoxic regions of gastric cancer and evaluated its impact on the TME and immunotherapy response. The nanodrug efficiently delivered TH-302 to hypoxic tumor zones, inducing selective cytotoxicity, reducing hypoxia, and remodeling the TME toward a more immune-supportive state. In murine models, combining the TH-302 nanodrug with PD-1 blockade significantly enhanced antitumor efficacy, leading to greater tumor regression and prolonged survival compared to either treatment alone (306).

## 6 Future perspectives

As our understanding of tumor biology deepens, it is increasingly evident that hypoxia is not a uniform phenomenon but a heterogeneous and dynamic feature of the TME. Different tumor types—and even regions within the same tumor—can display variable degrees of hypoxia and divergent metabolic adaptations (13). This heterogeneity presents both a challenge and an opportunity. One of the critical future directions lies in deciphering the molecular, metabolic, and spatial signatures that define hypoxia responses across diverse tumor contexts. Integrating these signatures into clinical practice could enable the development of hypoxia based stratification tools to identify patients who would benefit most from hypoxia-targeting therapies. This precision medicine approach could enhance treatment efficacy while minimizing off-target effects (307–310).

Another promising area involves combinatorial therapeutic strategies. Targeting hypoxia in isolation may yield limited benefits due to compensatory survival pathways (149, 217); however, combining hypoxia-targeted therapies with existing modalities such as immunotherapy, radiotherapy, or anti-angiogenic agents may offer synergistic effects (311–313). For instance, hypoxia-induced immunosuppression could potentially be reversed through co-administration of immune checkpoint inhibitors (313), while radiotherapy, which is less effective in hypoxic regions, may regain potency when combined with agents that reoxygenate tumors or sensitize hypoxic cells (314). Similarly, disrupting the aberrant vasculature of tumors with anti-angiogenic agents, in tandem with hypoxia-activated prodrugs, may lead to improved drug delivery and therapeutic outcomes (311, 315).

Technological advancements are also poised to revolutionize how we study and target hypoxia in cancer. Emerging tools such as single-cell metabolomics, spatial transcriptomics, and spatial metabolomics offer unprecedented resolution for analyzing the cellular and metabolic landscapes of the TME (316–318). Spatial metabolomics, in particular, integrates imaging mass spectrometry with molecular profiling to visualize metabolite distributions within intact tumor tissues, enabling the mapping of metabolic gradients associated with oxygen deprivation and therapy resistance. These technologies can unravel the spatial heterogeneity of hypoxia, delineate how individual cells adapt metabolically, and identify previously unrecognized subpopulations that may drive resistance to therapy (316, 319).

Coupled with artificial intelligence (AI) and machine learning (ML) algorithms, these multi-dimensional datasets can be mined for predictive biomarkers, therapeutic targets, and response indicators (318, 320). AI-driven hypoxia imaging using advanced radiomic and deep-learning models applied to MRI, PET, or multiparametric CT scan can non-invasively quantify hypoxic burden, monitor temporal changes during therapy, and guide adaptive treatment strategies (321–323). Integrating AI-driven spatial omics and imaging into clinical workflows holds the potential to transform hypoxia assessment from a static pathological finding to a dynamic, patient specific diagnostic tool. Together, these innovations pave the way toward next generation precision oncology, where real time hypoxia profiling informs individualized therapeutic interventions and improves clinical outcomes.

## 7 Conclusion

Hypoxia is a defining hallmark of the tumor microenvironment and a central orchestrator of tumor progression. It is not merely a byproduct of rapid tumor growth but a dynamic driver of metabolic and molecular reprogramming. Through stabilization of hypoxia-inducible factors (HIF-1α and HIF-2α), hypoxia activates extensive transcriptional programs that enable tumor cells to adapt to oxygen and nutrient deprivation. These programs promote a metabolic shift toward glycolysis, enhance glucose uptake and lactate production, modulate mitochondrial respiration, increase glutamine dependency, and stimulate lipid biosynthesis and storage. Concurrently, hypoxia regulates redox homeostasis, autophagy, angiogenic signaling (including VEGF induction), epithelial–mesenchymal transition, stemness, and extracellular matrix remodeling—collectively fostering invasion, metastasis, immune evasion, and resistance to radiotherapy, chemotherapy, and immunotherapy.

Importantly, hypoxia extends its influence beyond cancer cells to reshape the broader tumor ecosystem. It reprograms cancer-associated fibroblasts, endothelial cells, tumor-associated macrophages, myeloid-derived suppressor cells, and regulatory T cells, establishing a metabolically cooperative and profoundly immunosuppressive microenvironment. Hypoxia-induced acidosis and lactate accumulation further dampen cytotoxic T-cell and NK-cell function, while aberrant angiogenesis perpetuates perfusion defects and sustained oxygen gradients, reinforcing a vicious cycle of adaptation and selection.

Targeting the hypoxia–metabolism axis therefore represents a compelling therapeutic strategy. As highlighted in the review, approaches under investigation include hypoxia-activated prodrugs, HIF inhibitors, anti-angiogenic therapies, and metabolic enzyme modulators targeting glycolysis, glutaminolysis, and lipid metabolism. Although several agents have entered clinical evaluation, translation into durable clinical benefit remains challenging due to intratumoral heterogeneity, temporal fluctuations in oxygenation (cycling hypoxia), and adaptive resistance mechanisms that bypass single-pathway inhibition. Thus, a uniform therapeutic approach is unlikely to be effective.

Future progress will depend on integrating hypoxia signatures, metabolic profiling, and real-time oxygen mapping into precision oncology frameworks. Advanced methodologies—such as single-cell multi-omics, spatial transcriptomics, and functional hypoxia imaging—offer unprecedented resolution of tumor oxygen landscapes and metabolic dependencies. Rational combination strategies that concurrently target hypoxia signaling, metabolic vulnerabilities, angiogenesis, and immune checkpoints may ultimately overcome compensatory adaptations. In conclusion, hypoxia is not a passive consequence of tumor expansion but a dynamic, actionable vulnerability. Exploiting the hypoxia-driven metabolic circuitry with context-specific and biomarker-guided interventions holds substantial promise for improving therapeutic durability and clinical outcomes in cancer.
